# Structural basis of allosteric regulation of Tel1/ATM kinase

**DOI:** 10.1038/s41422-019-0176-1

**Published:** 2019-05-16

**Authors:** Jiyu Xin, Zhu Xu, Xuejuan Wang, Yanhua Tian, Zhihui Zhang, Gang Cai

**Affiliations:** 1 First Affiliated Hospital of USTC, School of Life Sciences, University of Science and Technology of China, Hefei, 230027 Anhui China; 2 Hefei National Laboratory for Physical Sciences at Microscale and CAS Center for Excellence in Molecular Cell Science, Chinese Academy of Sciences, Hefei, 230026 Anhui China; 3 College of Life Science and Technology, Huazhong University of Science and Technology, Wuhan, 430074 Hubei China

**Keywords:** Cryoelectron microscopy, DNA damage response

## Abstract

ATM/Tel1 is an apical kinase that orchestrates the multifaceted DNA damage response. Mutations of ATM/Tel1 are associated with ataxia telangiectasia syndrome. Here, we report cryo-EM structures of symmetric dimer (4.1 Å) and asymmetric dimer (4.3 Å) of *Saccharomyces cerevisiae* Tel1. In the symmetric state, the side chains in Tel1 C-terminus (residues 1129–2787) are discernible and an atomic model is built. The substrate binding groove is completely embedded in the symmetric dimer by the intramolecular PRD and intermolecular LID domains. Point mutations in these domains sensitize the *S. cerevisiae* cells to DNA damage agents and hinder Tel1 activation due to reduced binding affinity for its activator Xrs2/Nbs1. In the asymmetric state, one monomer becomes more compact in two ways: the kinase N-lobe moves down and the Spiral of α-solenoid moves upwards, which resemble the conformational changes observed in active mTOR. The accessibility of the activation loop correlates with the synergistic conformational disorders in the TRD1-TRD2 linker, FATC and PRD domains, where critical post-translational modifications and activating mutations are coincidently condensed. This study reveals a tunable allosteric network in ATM/Tel1, which is important for substrate recognition, recruitment and efficient phosphorylation.

## Introduction

The genome is constantly under assault by environmental agents, such as exposure to irradiation, chemical agents and ultraviolet light (UV), as well as endogenous agents, such as free radicals generated during normal metabolic processes. Cells have evolved surveillance mechanisms that monitor genomic lesions and activate various DNA damage responses, including cell cycle arrest and transcriptional induction of DNA repair genes^1^. In eukaryotes, this surveillance mechanism is called the DNA damage checkpoint. The checkpoint signals are initiated through two critical protein kinases: ataxia-telangiectasia mutated (ATM) and ATM-Rad3-related (ATR)^1,2^. ATM and ATR, master regulators of the DNA damage response, are highly conserved among eukaryotes. In budding yeast, Tel1 and Mec1 correspond to ATM and ATR, respectively^3^.

ATM primarily responds to DNA double-strand breaks (DSBs), whereas ATR is activated by DSBs as well as various types of DNA replication stress. Once activated, ATR and ATM phosphorylate a multitude (> 700) of substrates, such as p53 and checkpoint kinases Chk1 and Chk2, which are involved in cell cycle control, DNA repair, cell survival and other cellular processes^4^. These ATM and ATR-dependent phosphorylation events are essential to arrest cell cycle for DNA repair or to induce apoptotic cell death. Human ATM congenital deficiency results in ataxia telangiectasia^5^, a rare autosomal recessive disorder characterized by progressive cerebellar ataxia, neurodegeneration, radiosensitivity, checkpoint defects, genomic instability and predisposition to cancer.

ATM/Tel1 belongs to the evolutionarily conserved phosphatidylinositol-3-kinase-related protein kinase (PIKK) family. There are six members in the family, and each plays a pivotal role in controlling cellular homeostasis, including the DNA damage response (ATM, ATR, DNA-PKcs), cell growth (mTOR), mRNA decay (SMG1) and transcriptional regulation (TRRAP)^6^. The PIKKs share a highly conserved C-terminal FAT/kinase/FATC domain architecture^7,8^ and an extended N-terminal α-solenoids with lower sequence similarity^9–11^. The intrinsically active conformation of the kinase domain and restricted access of the active site are general features of the PIKKs^9,10,12–14^. Due to their diverse functions, sophisticated regulatory mechanisms are required to control the kinase activities of the PIKKs to adapt to particular pathways^6^. Under normal cellular conditions, ATM is inactive and exists in a homodimeric form. Upon DSB formation, ATM homodimers quickly undergo intermolecular autophosphorylation and are transformed into monomers after recruitment to DSB sites to fully activate their kinase activity^15^.

Given the central role of ATM in genome integrity and human disease, it is essential to understand the mechanism of its regulation. In particular, molecular and structural insights into ATM are critical to facilitate the design of therapeutic agents targeting the kinase^16^. However, due to their large size (300–500 kDa) and structural complexity, obtaining high-resolution structures of the PIKKs has remained a challenge. Cryo-EM has been a key contributor to our understanding of the architecture of ATM kinase. For instance, an 8.7 Å cryo-EM structure of yeast ATM/Tel1 and an intermediate resolution of human ATM with an overall resolution of 4.7 Å and 7.8 Å of closed and open conformers were reported^13,17^. Unfortunately, the resolutions of reported reconstructions were not high enough to allow residue identification and atomic model building, limiting our understanding of intramolecular and intermolecular interactions of ATM kinase necessary for DNA damage response.

Here we report a 4.1 Å cryo-EM structure of symmetric dimer and a 4.3 Å structure of asymmetric dimer of * Saccharomyces cerevisiae* ATM/Tel1. Determination of the structures in different conformational states offers us the unique opportunity to characterize the structural elements responsible for the long-range allosteric substrate recruitment and activation of ATM kinase.

## Results

### Overall structure of symmetric Tel1 dimer

**Fig. 1:**
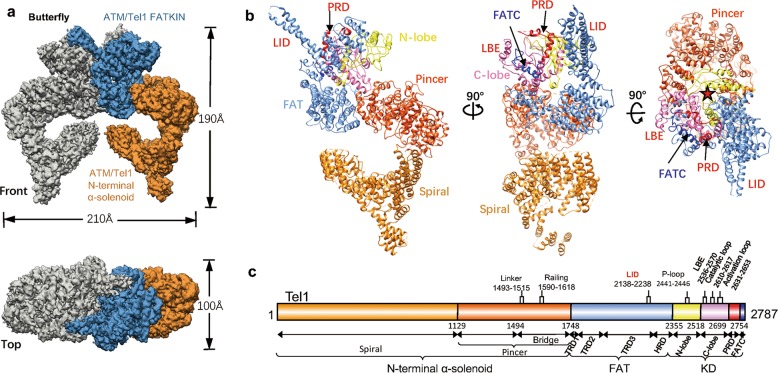
Cryo-EM reconstruction of Tel1 symmetric dimer. **a** Front and top views of the density map of Tel1 symmetric homodimer (named as Butterfly conformer). One monomer is color-coded by domain assignment: FATKIN in blue and α-solenoid in orange. The other monomer is shown as a solid gray surface. **b** Three views of the ribbon diagram model of the Tel1 Butterfly monomer. The N-terminal α-solenoid of Tel1 is colored in orange (Spiral) and dark orange (Pincer), FAT in cornflower blue, kinase N-lobe in yellow, C-lobe in hot pink, PRD in red and the FATC in blue. Each successive view is rotated as indicated. The red stars highlight the activation site. **c** A schematic representation highlighting the functional domains of Tel1. Three units of Tel1: N-terminal α-solenoid and Spiral, Pincer and Bridge region, FAT domain (TRD1, 2, 3 and HRD), kinase and PRD/FATC. The flexible regions controlling the Bridge and loops critical for ATM/Tel1 activity are shown above the schematic

The endogenously purified *S. cerevisiae* Tel1 displays basal kinase activity, which could be stimulated by incubation with specific activator Xrs2, the homolog of human Nbs1 (Supplementary information, Fig. S1). Two Tel1 molecules are juxtaposed in a side-by-side fashion, such that the complex has a butterfly-like dimeric architecture, which is similar to that of the Mec1-Ddc2^14^ (Fig. 1a; Supplementary information, Fig. S2). Interestingly, the Tel1 homodimer can either be symmetric (Fig. 1a) or asymmetric (Fig. 4a). First, we determined 4.1 Å Tel1 structure in the symmetric dimer state with a two-fold rotational (C2) symmetry (Supplementary information, Figs. S3, S4 and Table S1). Most of the side chains are discernible in the Pincer-FAT-KD-PRD-FATC domains, which allow us to build an atomic model (Supplementary information, Figs. S6 and S7). In contrast, the side chains of the 4.7 Å structure of human ATM are invisible^17^.

The Tel1 kinase domain is bilobal and resembles a canonical protein kinase. The ATP and the protein substrate simultaneously bind in the cleft formed between the N-terminal lobe (N-lobe) and larger C-terminal lobe (C-lobe). The active site is deeply recessed, which is generally in the intrinsically active conformation among PIKKs^6,10,12,14^. Several unique structural insertions in the C-lobe of the Tel1 kinase domain, such as the α-helical LBE (34 residues), the PRD (PIKK regulatory domain, 55 residues) and the FATC (FAT at C-terminus, 33 residues), encircle the active site elements, the activation and catalytic loops (Fig. 1b, c and 4a, b).

Immediately preceding the KD is an array of helical repeats constituting the FAT domain, which extends toward the N-terminal α-solenoid. The FAT domain intimately wraps around ~50% of the kinase domain, which has been suggested to regulate the binding of kinase substrates or regulators^6^. The N-terminal α-solenoid, adopting a highly sinuous super helical structure, could be morphologically segmented as two regions, Pincer (residues 1,129–1,747) and Spiral (residues 1–1,128) (Fig. 1b, c). The Pincer extends two arms cradling the catalytic core on top. Especially, one of the two arms corresponding to the Bridge (residues 1,497–1,747) is also conserved in ATR^14^ and TOR^18,19^.

### Interactions between FAT and kinase domain

The Tel1 FAT domain can be divided into four subdomains: tetratricopeptide repeat domains TRD1 (α1–α3, residues 1,747–1,788), TRD2 (α4–α11, residues 1,789–1,937), and TRD3 (α12–α22, residues 1,938–2,240), and HEAT repeat domain HRD (α23-α28, residues 2,241–2,356). The architecture of the FAT domain is highly conserved among ATM, ATR, mTOR and DNA-PKcs (Supplementary information, Figs. S8 and S9). Within the TRD3 region, the conserved ATM protrusion (corresponding to the α21 and α22), which was named as LBE-interacting domain (LID), tightly packs against the LBE of the other monomer at the Tel1 homodimer interface^13^ (Fig. 1b).

**Fig. 2:**
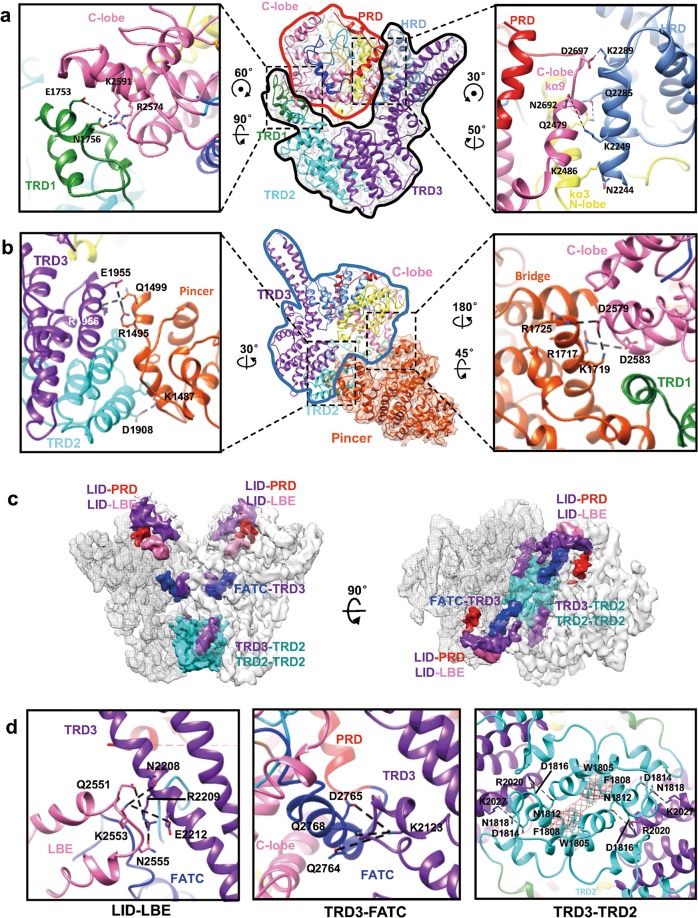
Details of the intramolecular and intermolecular interfaces. **a** Interactions between FAT and kinase domain. The cryo-EM density is shown as a translucent surface and fitted with the ribbon diagram model of the FATKIN (middle panel). The red contour denotes the kinase domain while the black contour highlights the boundary of FAT domain. The left panel shows the detailed interactions between the TRD1 and the kinase C-lobe. Dashed black lines denote potential salt bridges between Glu^1753^ (TRD1) and Lys^2591^ (C-lobe), and Asn^1756^ (TRD1) and Arg^2574^ (C-lobe). The right panel shows the interactions of HRD with the kinase N- and C-lobes. Dashed black lines denote potential salt bridges between Lys^2289^ (HRD) and Asp^2697^ (C-lobe), Gln^2285^ (HRD) and Gln^2479^ (N-lobe), Lys^2249^ (HRD) and Asn^2692^ (C-lobe), and Asn^2244^ (HRD) and Lys^2486^ (C-lobe). Each view is rotated as indicated. **b** Interactions between Pincer and FATKIN domain. The cryo-EM density of the pincer region is shown as a translucent surface and fitted with the ribbon diagram model. The blue contour highlights the FATKIN region. The left panel shows the detailed interfaces between the Pincer and the TRD2/TRD3. Dashed black lines denote potential salt bridges between Arg^1956^ (TRD3) and Gln^1499^ (Pincer), Glu^1955^ (TRD3) and Arg^1495^ (Pincer), and Asp^1908^ (TRD2) and Lys^1487^ (Pincer). The right panel shows that the Bridge of the Pincer and C-lobe make multiple contacts. Dashed black lines denote potential salt bridges between Asp^2579^/Asp^2583^ in C-lobe and Arg^1725^/Arg^1717^/Lys^1719^ in the Bridge region. **c** Cryo-EM structure of Tel1 symmetric FATKIN dimer highlighting the dimer interfaces. Two monomers of Tel1 are displayed as surface and mesh, respectively. **d** Enlarged view of the intermolecular LID-LBE, TRD3-FATC and TRD2-TRD2/TRD3-TRD2 interfaces. The potential hydrogen bonds are denoted by black dashed lines. The domain organization is color-coded: α-solenoid in orange, TRD1 domain in forest green, TRD2 domain in cyan, TRD3 domain in purple, HRD domain in cornflower blue, N-lobe in yellow, C-lobe in hot pink, PRD in red and FATC in blue

The Tel1 FAT domain makes multiple interactions with the kinase domain via its two extremely conserved segments HRD and TRD1 among PIKKs^12^ (Fig. 2a). TRD1 tightly binds to the base of LBE via the hydrogen bonds and electrostatic interactions (Glu^1753^-Lys^2591^ and Asn^1756^-Arg^2574^) (Fig. 2a, left panel; Supplementary information, Fig. S10a). HRD makes multiple contacts with kinase C-lobe (via kα9) through a hydrogen bond network between Lys^2289^-Asp^2697^ and Lys^2249^-Asn^2692^ and with N-lobe (mainly via kα3) through the interaction between Gln^2285^-Gln^2479^ and Asn^2244^-Lys^2486^ (Fig. 2a, right panel; Supplementary information, Fig. S10b). Strikingly, ATM point mutation R2849P (corresponding to Lys^2591^ in Tel1) at the LBE-TRD1 interface and R2486P (corresponding to Lys^2249^ in ScTel1) at the N-lobe-HRD interface is directly associated with T cell prolymphocytic leukemia^20^ and A-T disease^21^, respectively. The pathogenic mechanism of the R2849P and R2486P mutations could be disrupting the critical TRD1-LBE or HRD-N-lobe interfaces and destabilizing ATM.

### Interactions between Pincer and FATKIN domain

The N-solenoids of ATM/Tel1^13,17^ and ATR/Mec1^14^ share five structural modules, the Spiral, Pincer, Bridge, Railing and Linker, which are organized in the same way. The Pincer extends two arms cradling the catalytic core (Fig. 2b, middle panel). One arm tightly contacts with the TRD2 and TRD3 domains via the hydrogen bonds and electrostatic interactions between Arg^1956^ (TRD3) and Gln^1499^ (Pincer), Glu^1955^ (TRD3) and Arg^1495^ (Pincer), and Asp^1908^ (TRD2) and Lys^1487^ (Pincer) (Fig. 2b, left panel; Supplementary information, Fig. S10c). The other arm corresponding to the Bridge directly interacts with the kinase C-lobe through the potential salt bridges between Asp^2579^/Asp^2583^ (C-lobe) and Arg^1725^/Arg^1717^/Lys^1719^ (Bridge) (Fig. 2b, right panel; Supplementary information, Fig. S10d).

Having a scaffolding function, the Bridge could play a critical role as a co-regulatory element of the kinase activity by positioning itself in front of the catalytic cleft, which is ready to recruit and position binding partners or substrates close to the active site. Consistently, the conserved Bridge domain critically regulates the kinase activity of PIKKs^14^, a feature shared by human ATR^22,23^ yeast Mec1^24^, human ATM^23,25^, DNA-PKcs^23,26^, and mTOR^19^. In particular, the mTOR Bridge domain is responsible for binding of Raptor, which directly interacts with the Tor signaling sequence (TOS) motif of substrates and regulators^27^. The same property of the Bridge might be expected for other PIKKs.

### Tel1 dimerization interfaces

The atomic model of Tel1/ATM (residues 1,129–2,787) allows us to take a closer examination of the molecular details of the dimerization interfaces. Unlike the Mec1-Ddc2 dimer architecture^14^, the bottom α-solenoid does not interact with each other in Tel1/ATM homodimer. The three intermolecular interfaces in the Tel1 homodimer are centered on the FATKIN (PIKK FAT and C-terminal kinase domain), which are LID-LBE/LID-PRD, TRD3-FATC and TRD2-TRD2/TRD2-TRD3 from top to bottom (Fig. 2c, d).

**Fig. 3:**
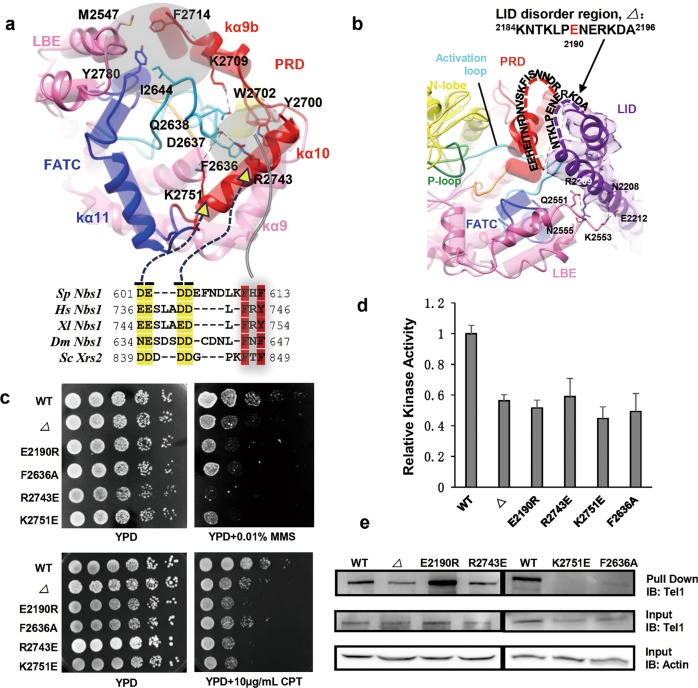
Modulation of the substrate-binding groove. **a** Superimposition of the structures of substrate-binding groove in human ATM (shown as transparent pipes, PDB ID: 5NP0) and yeast Tel1 (shown in solid ribbons), highlighting the interaction between PRD/FATC/LBE and activation loop. The side chains of residues mediating the critical interactions are denoted. Gray translucent regions highlight two hydrophobic pockets. Conserved sequence characteristics of the Xrs2/Nbs1 activation domain (AAD), which harbors two conserved aromatic residues (red) as well as one or two consecutive acidic patches (yellow). The possible functional interactions between extremely conserved residues of AAD and the residues of PRD immobilizing the activation loop are denoted. **b** Overlay of the structures of human ATM (shown as pipes, PDB ID: 5NP0) and yeast Tel1 (shown in ribbons), highlighting the intermolecular PRD-LID dimer interface. To highlight the LID domain from the other monomer, the cryo-EM density of LID is shown in transparent surface and fitted with ribbon model in purple. The sequence of the disordered regions in PRD and LID is labeled and connected by dashed lines. P-loop in green, catalytic loop in orange and activation-loop in cyan. **c** DNA damage sensitivity of Tel1 mutants: Δ (truncation of LID disordered region 2,184–2,196), E2190R, F2636A, R2743E and K2751E. Exponential yeast cultures were serially diluted and spotted onto YPD plates containing 0.01% MMS or 10 μg/mL CPT. **d** The in vitro kinase activity assay of Tel1 mutants using the ADP-Glo™ kit as described in Materials and Methods. *n* = 3. Results are represented as means ± SD. The equal amount of enzyme addition was adjusted by western blotting. **e** The pull-down assay of Tel1 mutants with Xrs2/Nbs1 in vitro. The lysates from the strains expressing wild-type and mutant Tel1 were precipitated with GST-Xrs2 beads and subjected to immunoblot with anti-PAP antibody. The actin is used for a loading control

The LID domain makes multiple interactions with the LBE through a hydrogen bond network, which involves Asn^2208^ (LID)-Lys^2553^ (LBE), Glu^2212^ (LID)-Lys^2553^ (LBE), Arg^2209^ (LID)-Gln^2551^ (LBE) and Arg^2209^ (LID)-Asn^2555^ (LBE) (Fig. 2d, left panel; Supplementary information, Fig. S11a). In addition, the structure discloses that PRD and LID domains are spatially close to each other (Fig. 2d, left panel and 3b). Both domains contain disordered fragment (^2716^EEHEITNFDNVSKFISNNDR^2735^ in PRD and ^2184^KNTKLPENERKDA^2196^ in LID) with complementarily charged residues, and may intertwine with each other (Fig. 3b).

The FATC domain contains a hydrophobic pocket essential for the substrate selectivity and could serve as a putative substrate docking site^28,29^. TRD3 directly interacts with the FATC helix kα11 via Lys^2123^ (α20), which makes a hydrogen bond network by contacting conserved Gln^2764^ (kα11), Asp^2765^ (kα11) and Gln^2768^ (kα11) (Fig. 2d, middle panel; Supplementary information, Fig. S11b). Through the TRD3-FATC interface, TRD3 may directly modulate substrate binding to the Tel1 active site.

The TRD2-TRD2 dimer interface is a conserved interface shared by Tel1 and Mec1^13,14^, which is stabilized by a hydrophobic interface between the conserved residues of the Trp1805 (α5) and Phe1808 (α5). In addition, several polar or charged residues at the TRD2 and TRD3 regions stabilize the TRD2-TRD2 contacts (Fig. 2d, right panel; Supplementary information, Fig. S11c), which are Arg^2020^ and Lys^2027^ in α14, and Asn^1812^, Asp^1814^, Asp^1816^ and Asn^1818^ in α5. Interestingly, TRD3 domain straddles all the three dimer interfaces and critically contributes to the stability of Tel1/ATM homodimer (Fig. 2c, d). Therefore, TRD3 could play a critical role in the allosteric regulation through synergistically modulating all the dimerization interfaces and the dimer-monomer transition.

### Modulation of the substrate binding groove

The structures of yeast Tel1 and human ATM are highly conserved; not only the ordered secondary structures closely resemble each other, but also the flexible loops and disordered fragments harbor similar architectures (Fig. 3a, b; Supplementary information, Fig. S9a). The Tel1 and ATM harbor the signatures of an active conformation. The elements crucial for catalysis are ordered in the structure, including the activation loop, catalytic loop, P-loop (Fig. 3a, b). The kα10 and kα9b helices of PRD intimately pack against the activation loop and cap the catalytic site (Fig. 3a). The Tyr^2700^ and Trp^2702^ residues in kα10 directly contact the exposed Phe^2636^ of the activation loop by hydrophobic interactions. Furthermore, the Asp^2637^ and Gln^2638^ in the N-terminal activation loop interact with Lys^2751^/Arg^2743^ in kα10 by electrostatic interactions and Lys^2709^ in kα9b by hydrogen bonds, respectively (Fig. 3a; Supplementary information, Fig. S12b). Lys^3016^ (corresponding to Lys^2751^ in yeast Tel1) acetylation is a priming step for ATM activation after DSB^30^. The acetylation may destabilize PRD’s immobilization effect on activation loop, facilitating ATM activation.

The FATC domain is composed of a pair of helices, kα11 and a three-segmented kα12 (a/b/c) at the end of the kinase C-lobe. The Tel1 activation loop uses its C-terminal ^2644^IPELVPFRL^2652^ motif to directly interact with ^2780^YMAWSPFY^2787^ of kα12b helix, stacking its Ile^2644^ perpendicular to Tyr^2780^ (kα12) (Fig. 3a; Supplementary information, Fig. S12a). The kα12 sequence is highly conserved among the mTOR (^2542^YIGWCPFW^2549^) and ATM/Tel1 (^2780^YMAWSPFY^2787^) orthologues. Similar to Tel1, the mTOR activation loop (^2371^FPEKIPFRL^2379^) also directly packs against the FATC kα12 through the hydrophobic interaction between Phe^2371^ (activation loop) and Tyr^2542^ (kα12)^12^. In addition, the last 10 amino acids (kα12) of human ATM are essential for oxidation-mediated activation^31^. These observations consistently suggest that the FATC kα12 could directly affect the structure of the activation loop.

Together with the helices kα9b and kα10 (PRD), the kα11 and kα12 (FATC) serve as a structural framework for the Tel1 kinase domain, encircling the activation and catalytic loops. The Phe^2714^ (PRD), Met^2547^ (LBE), Tyr^2780^ (FATC) and Ile^2644^ (activation loop) form a hydrophobic patch and enclose the substrate-binding groove (Fig. 3a). The LID domain tightly packs against both the intermolecular PRD and LBE (Fig. 3b), further blocking substrate entry from the above. Thus, the substrate-binding groove is fully restricted in the Tel1 homodimer in all directions, which is completely embedded in the symmetric dimer (Fig. 3a, b). The PRD, FATC, LBE and LID domains potentially allow tight regulation of the ATM/Tel1 kinase activity by redundantly regulating the binding of substrates or regulators to the active site.

We then characterized the regulatory functions of the PRD and LID domains by introducing substitution or truncation mutations (Supplementary information, Fig. S13). Five mutants in the two domains, E2190R, F2636A, R2743E, K2751E and Δ, which is a truncation of Tel1 LID disordered region 2,184–2,196, did not affect the Tel1 expression level. However, these mutations impaired the cellular response to DNA damage, as revealed by enhanced sensitivity of yeast cells to various chemotherapeutic drugs including MMS, CPT and HU (Fig. 3c; Supplementary information, Fig. S14), accompanied by decreased kinase activities toward the exogenous substrate nucleosome and protein activator Xrs2 (Fig. 3d; Supplementary information, Fig. S15). Further studies showed that four of the five mutants interact with the C-terminus of Xrs2 less efficiently than wild-type Tel1 to various degrees; in particular, the K2751E (PRD kα10) and F2636A (activation loop) mutants almost abolished the interaction with Xrs2 (Fig. 3e; Supplementary information, Fig. S16). The decreased affinity between Tel1 and Xrs2 could explain that these mutations led to enhanced sensitivity to DNA damage and reduced kinase activity.

Xrs2/Nbs1 is an essential protein to mediate Tel1/ATM association with chromatin and activation upon induction of DSBs. Previous model suggests that the C-terminus of Xrs2/Nbs1 and the α-solenoid of Tel1/ATM interact with each other^32,33^. Recently, it was shown that the Tel1 FATC also mediates Tel1-Xrs2 interaction and contributes to its localization to DNA ends and target protein recognition^34^. Similar to the Mec1/ATR activation domain (AAD)^14,35^, the Xrs2/Nbs1 AAD also harbors two extremely conserved aromatic residues, along with one or two highly conserved and consecutive pairs of acidic residues, which are critical for Tel1/ATM interaction and activation^33,36^ (Fig. 3a, bottom panel). The aromatic residues and two pairs of acidic residues in AADs could target Tyr^2700^/Trp^2702^ and Lys^2751^/Arg^2743^/Lys^2709^ in Tel1/ATM PRD domain and destabilize the PRD’s immobilization effect on activation loop. Tel1/ATM and Mec1/ATR probably share the conserved activation mechanism^14^, by which the AAD targets the PRD and relieves PRD inhibition on the activation loop and substrate-binding groove (Fig. 3a, bottom panel).

### Overall structure of asymmetric Tel1 dimer

**Fig. 4:**
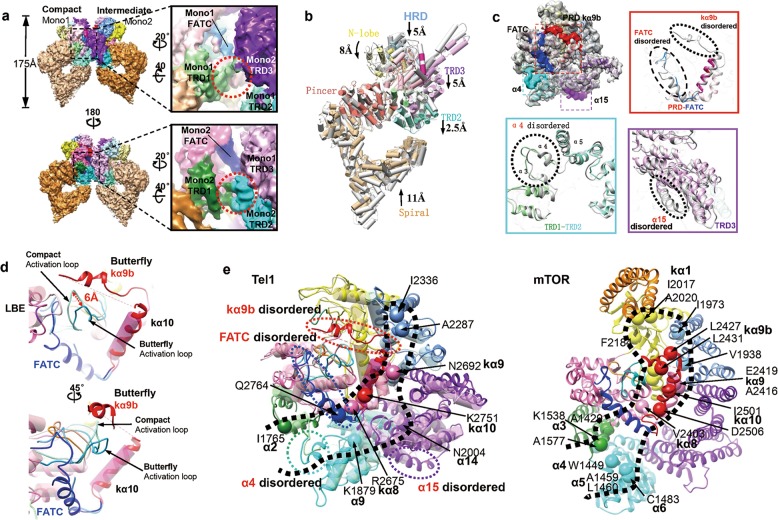
Structures of the asymmetric Tel1 dimer. **a** Two views of the 3D reconstruction of asymmetric Tel1 homodimer. The two monomers from the asymmetric dimer show substantial structural differences (Mono1 is named as Compact and Mono2 is named as Intermediate). The domain organization is color-coded: α-solenoid in orange, TRD1 domain in forest green, TRD2 domain in cyan, TRD3 domain in purple, HRD domain in cornflower blue, N-lobe in yellow, C-lobe in hot pink, PRD in red and FATC in blue. The Compact monomer is in less saturated color than the Intermediate monomer. The right panel denotes the substantial variations in the TRD1-TRD2 linker and FATC regions, which is largely disordered in the Compact conformer. The red circles highlight the differences in the TRD1-TRD2 linker. **b** Comparison of the overall Tel1 structures of the Compact (domain color-coded as **a**) and Butterfly (gray pipes) conformers. In the comparison, the Pincer and C-lobe are superimposed with each other, and the positions of the N-lobe, FAT and Spiral are compared. The motion ranges of the N-lobe, HRD, TRD2, TRD3 and Spiral are denoted. **c** The four disordered fragments in the Compact monomer highlighted on the Butterfly structure and close-up views of each disordered region (α4 in TRD2, α15 in TRD3, kα9 in PRD and kα11 and kα12 in FATC). The disordered fragment of Compact conformer (color-coded as **a**) and the corresponding region of Butterfly conformer (gray) are superimposed with each other. **d** Overlay of the structures of substrate-binding groove in Butterfly conformer (shown in ribbons) and that in Compact conformer (shown as transparent pipes in less saturated color), highlighting that the activation loop moves outwards by ~6 Å in Compact monomer (denoted by a red arrow). **e** The Tel1/ATM activating mutations are mapped onto the structures of the superimposed substrate-binding grooves of the two conformers (left panel). The Butterfly and Compact conformers are shown in ribbons and transparent pipes, respectively. The activating mutations of mTOR are mapped on the crystal structure (PDB ID: 4JSV, right panel). The activating mutations are shown as large spheres (labeled, with structural elements in bold). The black dashed contours highlight a good correspondence of Tel1/ATM and mTOR activating mutations along a putative substrate-recruitment channel

Besides the 4.1 Å cryo-EM structure of symmetric dimer, we also determined a 4.3 Å structure of asymmetric dimer of Tel1/ATM, which allows to trace the course of the polypeptide. The asymmetric structure is probably representative of the pathway toward activation, which is significantly promoted by known Tel1 activators such as MRX complex or Xrs2 C-terminus (Supplementary information, Fig. S17). Determining the Tel1/ATM structures in two dimeric conformations offers the opportunity to characterize the structural elements responsible for the unique long-range allosteric substrate recruitment and activation of ATM/Tel1 kinase. In the asymmetric state, one of the monomers become more compact than the other, which are named as ‘Compact’ and ‘Intermediate’ conformers, respectively (Fig. 4a; Supplementary information, Figs. S3 and S5. The significant structural differences of the two monomers exist in the TRD1-TRD2 linker region and FATC domains, which seems to outline a putative channel leading to the substrate-binding groove.

We next compared the Tel1 structures of the Compact and Butterfly conformers. The Pincer and C-lobe are superimposed with each other, and the positions of the FAT, N-lobe and Spiral are compared. In the Compact Tel1, the FAT and kinase N-lobe move down by ~5–8 Å, whereas the Spiral of α-solenoid moves upwards by ~11 Å relative to their positions in the Butterfly conformer (Fig. 4b). The overall height has been reduced from 190 Å (Butterfly) to 175 Å (Compact). The activated mTOR adopts similar conformational changes and the overall structure becomes more compact^27^. It is well-known that motion of the N-lobe generally leads to an active and closed conformation, which facilitates ATP and substrate binding to the active site inside the central cleft. These observations suggest that the Compact conformer is closer to the activated Tel1 than the Butterfly conformer.

The kα11 and kα12 (FATC) are partially disordered, while the α4 (TRD2), α15 (TRD3) and kα9b (PRD) are almost fully disordered in the Compact conformer (Fig. 4c; Supplementary information, Fig. S18). The α4 of TRD2 and α15 of TRD3 are close to each other in the Tel1 homodimer interface (Supplementary information, Fig. S19). The synergistic conformational disorder of α4 and α15 would facilitate enlarging the putative channel leading to the substrate-binding groove. As the PRD and FATC become disordered, their inhibition on the activation loop is at least partially relieved, which can now move outside of the substrate-binding groove by ~6 Å (Fig. 4d). The activation loop generally undergoes substantial conformational changes during catalysis that are intimately tied to activation^28^. These coordinated conformational changes of the FAT, FATC, PRD and the activation loop could expose the substrate binding site and facilitate Tel1 activation.

### The putative channel for substrate recruitment

**Fig. 5:**
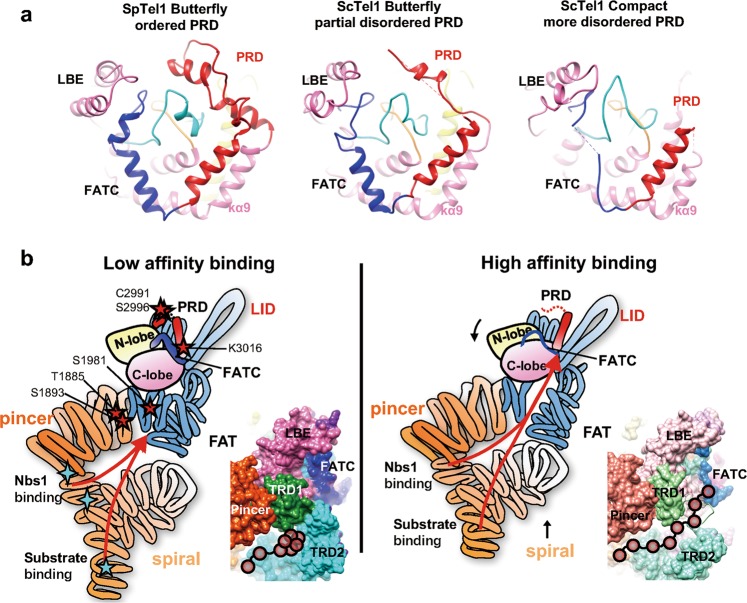
Substrate recruitment mechanism of ATM/Tel1 kinase. **a** The Tel1 substrate-binding groove in different conformational states. Left, fully ordered PRD and FAT domains of *S. pombe* Butterfly conformer tightly restrict the activation loop. Middle, the partially disordered PRD and FATC of *S. cerevisiae* Butterfly conformer makes the activation less restricted. Right, the increasingly disordered PRD and FATC in the Compact conformer facilitate exposing the activation loop and substrate-binding groove. **b** Structural schematic and surface presentation of the putative substrate-recruitment channel in different functional states, highlighting coordinated conformational changes in the α-solenoid, FAT, kinase N-lobe, PRD, and FATC. TRD1-TRD2 linker, FATC and PRD could act as the gateways controlling substrate access to the active site. In the Butterfly conformer (left panel), the ordered TRD2, FATC and PRD block substrate access. The disordered TRD2, FATC, and PRD in the Compact conformer (right panel) could facilitate substrate recruitment into active site. The Tel1/ATM critical post-translational modification sites (red stars) and substrate/activator binding sites (cyan stars) are denoted in the left panel

The intrinsic flexibilities of the TRD1-TRD2 linker, FATC and PRD suggest a putative channel into the Tel1 substrate-binding groove (Fig. 4e, left panel). In the Butterfly conformation, the fully ordered α4 (TRD2), α15 (TRD3), kα11 (FATC), kα12 (FATC) and kα9b (PRD) fully restrict the substrate-binding groove. However, the disordered fragments in the Compact monomer release these constraints, leaving sufficient space for a peptide substrate to bind (Fig. 4e, left panel). Furthermore, the activating mutations in Tel1/ATM^37^ and mTOR^38,39^ are coincidentally clustered around the substrate-binding groove, condensing in the TRD1-TRD2, FATC, PRD and HRD regions (Fig. 4e). The coincidental distribution of activating mutation sites of Tel1/ATM and mTOR also outlines the putative substrate recruitment channel. Moreover, the channel is under tight regulation by autophosphorylations of Thr^1885^ and Ser^1893^ in the α-solenoid and Ser^1981^ in the TRD1^40^, which are in close proximity to the linker region of TRD1-TRD2 (Fig. 5b). Furthermore, the other post-translational modifications critical for ATM activation, including Ser^2996^ autophosphorylation^15,40^, Cys^2991^ disulfide bond formation^31^ and Lys^3016^ acetylation^41^, are highly condensed in the PRD domain (Fig. 5b). The NuA4/Tip60 acetyltransferase complex needs to bind to the FATC^8^ and acetylates the PRD of ATM^41^. These concentrated post-translational modifications, activating mutation distribution and conformational modulation in the TRD1/TRD2, PRD and FATC collectively highlight the tunable substrate recruitment channel, which is under rigorous regulation in gating substrate delivery to Tel1 active sites.

## Discussion

The study discloses that the PRD (kα9b and kα10) and FATC (kα11 and kα12) encircle the Tel1 active site elements, the activation and catalytic loops. In particular, the Phe^2714^ (PRD), Met^2547^ (LBE), Tyr^2780^ (FATC) and Ile^2644^ (activation loop) form a hydrophobic pocket and fully enclose the substrate-binding groove (Fig. 3a). Structural comparison of the Tel1/ATM, Mec1/ATR^14^, mTOR^12^ and DNA-PKcs^10^ suggests a conserved regulatory mechanism on the substrate-binding groove, where PRD and FATC critically control the substrate-binding groove by immobilizing the activation loop (Supplementary information, Fig. S20). We recently improved the published *S. pombe* Tel1 structure from 8.7 Å^13^ to 6.1 Å and found that it harbors fully ordered PRD and FATC (Fig. 5a, left panel; Supplementary information, Fig. S21). Comparing the fission yeast *S. pombe* reconstruction with the structures of budding yeast *S. cerevisiae* in two conformations, it is obvious that as the disorder degree of PRD and FATC is increased, the structural flexibility of the activation loop is also significantly enhanced. The activation loop could move outside of the substrate-binding groove to access substrates in the Compact conformer. Therefore, the conformations of PRD and FATC directly affect the structure of the activation loop.

To enable catalysis, the kinase activation loop should undergo substantial conformational change and needs to move outside of the substrate-binding groove to access substrates^28^. However, the Tel1/ATM activation loop in the Butterfly conformer is directly immobilized by PRD and FATC domains, which inhibit the substrate binding and enzyme activation by enclosing the substrate-binding groove (Fig. 3a). The intrinsic conformational flexibility of the PRD and FATC could at least partially relieve their inhibition on the activation loop and contribute to the basal Tel1/ATM kinase activity (Fig. 5b). The total relief of such inhibition critically relies on its specific activator Xrs2/Nbs1^42^. The extremely conserved aromatic residues and acidic patches in the Xrs2/Nbs1 AAD could specifically destabilize PRD-activation loop interfaces, contributed by the hydrophobic residues and basic patches of PRD (Fig. 3a). The direct supporting evidence comes from site-directed mutagenesis studies and pull-down assays. The conserved aromatic and acidic residues in Xrs2/Nbs1 AAD have been verified to be critical for binding to Tel1/ATM^33,36^. Our data suggest that the PRD K2751E mutation decreases Xrs2 binding by almost 100% (Fig. 3e). The AAD mutants and PRD mutants coincidently decrease the Xrs2/Nbs1-Tel1/ATM binding affinity, suggesting that AAD and PRD may directly interact. In addition, the Tel1 FATC also mediates Tel1-Xrs2 interaction and contributes to Tel1 localization and target protein recognition^34^. Accordingly, Xrs2/Nbs1 activates Tel1/ATM probably by means of destabilizing immobilization effect of PRD and FATC on activation loop.

The N-terminal a-solenoid of ATM/Tel1 has been identified to contain substrate and activator binding sites^33,36^ (Fig. 5b, left panel). It is intriguing how the substrate and activator bound to the bottom α-solenoid are recruited to the active site on the top. Since Xrs2/Nbs1 is also one of Tel1/ATM substrates^43,44^, it needs to be delivered to the upper active site. The substrates could access catalytic site through the disordered TRD1-TRD2 linker, FATC and PRD gateways. Consistently, the critical post-translational modifications and activating mutations are also condensing around these regions (Figs. 4e and 5b). The putative substrate-recruitment channel is probably responsible for the substrate recognition and delivery.

mTORC1 selects substrates through two independent substrate-recruitment sites: RAPTOR subunit and mTOR FRB domain^27^. The Tel1/ATM substrate-binding site in N-terminal α-solenoid could function equivalently to RAPTOR subunit of mTORC1, however, it still lacks the equivalent of FRB substrate-recruitment site. In the activated mTOR, the N-heat solenoid swings inwards making contacts with the FAT, which causes conformational changes in the kinase-proximal FAT portion and N-lobe domains, closing the catalytic cleft between the N- and C-lobes^27^. Similar conformational changes exist in the Compact Tel1, with the Spiral of α-solenoid moving upwards and kinase N-lobe moving down (Fig. 5b, right panel). These observed coordinated conformational changes of the α-solenoid, kinase-proximal FAT, PRD and FATC help deliver the substrate bound to the N-terminal α-solenoid into close proximity to the upper kinase active site. Accordingly, Tel1/ATM could critically rely on the tunable allosteric network in TRD1-TRD2, PRD, FATC for substrate recognition, recruitment and efficient phosphorylation.

## Materials and methods

### Purification of yeast ATM/Tel1

*S. cerevisiae* strain (*MATa TEL1-FLAG his3*Δ*1 leu2*Δ*0 met15*Δ*0 ura3*Δ*0*)^45^ was grown in YPD medium to the stationary phase. About 100 g cells were harvested, washed and re-suspended in extraction buffer (50 mM HEPES, pH 8.0, 300 mM KOAc, 0.5 mM EDTA, 5 mM β-ME, 10% (v/v) glycerol, 0.1% (v/v) NP-40 and protease inhibitors), and whole-cell extract was prepared as previously described^46^. The whole-cell extract was selectively precipitated in 30%–55% ammonium sulfate and re-suspended using 1× TEZ buffer (50 mM Tris-HCl, pH 7.5, 1 mM EDTA, 10 mM ZnCl_2_, 5 mM β-ME and protease inhibitors). After the suspension was clarified by centrifugation, the supernatant was incubated for 2 h at 4 °C with 1 mL of a 50% slurry of FLAG resin beads (GE Healthcare) that had been pre-equilibrated with 1× TEZ plus 250 mM ammonium sulfate. After incubation, the beads were washed with 50 mL of 1× TEZ plus 500 mM ammonium sulfate, followed by a second wash with 50 mL of 1× TEZ plus 50 mM ammonium sulfate. After equilibration of the column with 1× TEZ plus 100 mM ammonium sulfate, 10 mM 3× FLAG peptide (Sigma) was added to the resin beads and incubated for 1 h at 4 °C. The Tel1 fraction was then eluted with three column volumes of 1× TEZ plus 50 mM ammonium sulfate and the resulting aliquot was snap-frozen in liquid nitrogen and temporarily stored at −80 °C. For the next purification step, the FLAG elution fractions were diluted to 1× TEZ plus 50 mM ammonium sulfate, applied onto a Mono S column (GE Healthcare) and resolved over a 50–1,000 mM ammonium sulfate gradient. The Tel1 elution was flash-frozen in liquid nitrogen, and analyzed by SDS-PAGE and EM examination.

### Recombinant expression and purification of full-length and C-terminal Xrs2 (835–854 aa)

*S. cerevisiae* Xrs2 C-terminus (835–854 aa) was cloned into the expression vector pET22b and expressed in *E. coli* BL21 (DE3). After the culture reached a cell density of OD_600_ of 0.5–0.7, 0.5 mM IPTG was added to the culture for 4 h at 16 °C. The cells were harvested and re-suspended in binding buffer (20 mM Tris, pH 8.0, 500 mM NaCl, 5% glycerol, 10 mM DTT). The whole-cell extract was centrifuged at 16,000× *g* for 30 min at 4 °C. Then, the supernatant was incubated for 1 h at 4 °C with 1 mL Ni-NTA resin beads that had been pre-equilibrated with binding buffer. The beads were washed with 50 mL of binding buffer plus 20 mM imidazole, followed by a second wash with 50 mL of binding buffer plus 50 mM imidazole. Binding buffer plus 250 mM imidazole was added to the resin beads and incubated for 30 min at 4 °C. The Xrs2 fraction was then eluted with three column volumes of elution buffer and the resulting aliquots were snap-frozen in liquid nitrogen and stored at −80 °C.

*S. cerevisiae* Xrs2 was cloned into pGEX-6P-1 at *Bam*HI and *Xho*I sites, and transformed into the expression host *E. coli* BL21 (DE3). Cells were grown in LB broth until OD_600_ of ~0.4, and Xrs2 expression was induced by the addition of IPTG to a final concentration of 0.5 mM. Cells were further grown for 5 h at 16 °C, and harvested by centrifugation at 8,000× *g* for 5 min. The cell pellet was re-suspended in extraction buffer (50 mM HEPES, pH 8.0, 300 mM KOAc, 0.5 mM EDTA, 5 mM β-ME, 10% (v/v) glycerol, 0.1% (v/v) NP-40 and protease inhibitors). Cells were lysed by sonication and centrifuged at 16,000× *g* for 60 min. The cell lysate was selectively precipitated in 30%–55% ammonium sulfate and re-suspended using 1× TEZ buffer (50 mM Tris-HCl, pH 7.5, 1 mM EDTA, 10 mM ZnCl_2_, 5 mM β-ME and protease inhibitors). After the suspension was clarified by centrifugation, the supernatant was incubated for 2 h at 4 °C with 1 mL of glutathione sepharose (GE Healthcare). After incubation, the beads were washed with 50 mL of 1× TEZ plus 500 mM ammonium sulfate, followed by a second wash with 50 mL of 1× TEZ plus 50 mM ammonium sulfate, and 30 mM GSH was added to the resin beads and incubated for 1 h at 4 °C. For the next purification step, the Xrs2 elution fractions were applied onto a Mono Q column (GE Healthcare) in 1× TEZ plus 50 mM ammonium sulfate and resolved over a 50–1000 mM ammonium sulfate gradient. The fractions containing Xrs2 were combined and incubated with glutathione sepharose for 2 h at 4 °C. Then the beads were washed with 1× TEZ containing 500 mM ammonium sulfate, and 30 mM GSH was added to the resin beads and incubated for 1 h at 4 °C. The Xrs2 elution was flash-frozen in liquid nitrogen and analyzed by SDS-PAGE.

### Spot assay

Tel1 mutant strains used in the assay were generated in the background of W303-1a (*MATa ade2-1 ura3-1 his3-11,15 leu2-3,112 can1-100*) and N-terminally tagged with TAP. Yeast strains were grown overnight to stationary phase in YPD medium and diluted to OD_600_ of 0.8. Six-fold serial dilutions of yeast cultures were spotted on YPD plates that were supplemented with 2 mM HU or 10 μg/mL CPT, 0.01% MMS or treated with 100 J/m^2^ UV.

### Kinase activity assay

Kinase assay of the affinity-purified ATM/Tel1 was performed in a 10 μL volume of 50 mM HEPES, pH 8.0, 100 mM KOAc, 1 mM EDTA, 10% (v/v) glycerol, 10 μM ZnCl_2_, 0.5 mM DTT, 8 mM magnesium acetate (final concentration, including contributions made by protein storage buffers), 100 μM ATP with GST-Mek1(1–50 aa) as substrate^47^ at the indicated concentrations and 240 nM Xrs2(835–854 aa) as activator. Assays were started by addition of 5–10 nM Tel1, incubated at 30 °C for 2 h and stopped by addition of 10 µL ADP-Glo™ Reagent (Promega #V9101) and incubation at room temperature for 40 min. Kinase Detection Reagent (20 µL) was added to convert ADP to ATP by incubation at room temperature for 45 min, and then the luminescence was recorded immediately.

Kinase assays of the immunoprecipitated wild-type and mutant ATM/Tel1 were performed as described below. The wild-type and mutant Tel1 were immunoprecipitated with IgG sepharose (GE, Cat# 17096901) from the cell lysate untreated or treated with 0.01% MMS or 5 µM CPT. The kinase assays were performed in kinase buffer (10 mM HEPES, pH 7.4, 10 mM MnCl_2_, 10 mM MgCl_2_, 1 mM DTT, 100 µM ATP) containing purified full-length Xrs2 activator and nucleosome substrate. After 30 min of incubation at 30 °C, the reaction was stopped by addition of ADP-Glo^TM^ reagent. The kinase detection buffer was added to the mixture and incubated at room temperature for 45 min, and the luminescence was recorded immediately.

### Pull-down assay

The yeast cells expressing wild-type and mutant Tel1 were lysed in lysis buffer and the glutathione sepharose (GE, Cat# 17-5132-02) saturated with full-length Xrs2 protein was added to the lysates. After 2 h incubation at 4 °C, the beads were collected and washed 3 times with lysis buffer. The proteins were eluted with SDS sample buffer and boiled at 100 °C for 15 min. The eluted protein was analyzed with 8% SDS-PAGE, followed by immunoblot analysis using anti-PAP (Sigma, Cat# P1291) antibody.

### EM sample preparation, data collection and image analysis

The Mono S fraction was diluted 2–4 times (20 mM HEPES, pH 8.0, 40 mM KOAc, 5 mM MgCl_2_, 0.1% trehalose, 2 mM DTT, 0.01% NP-40) and applied to a carbon-coated 400-mesh Cu EM specimen grid freshly glow discharged. The grid was then preserved by staining with 0.75% (w/w) uranyl formate solution. Images were recorded at a magnification of 62,000× on a 4,096 × 4,096 CCD detector (FEI Eagle) with a Tecnai F20 electron microscope (FEI) operating at an acceleration voltage of 200 kV. Images were recorded by using low-dose procedures, at ~0.6–0.8 μm under focus. Two-fold pixel binning of the original CCD images resulted in a final pixel size of 3.54 Å per pixel.

The particles show a strongly preferred orientation upon adsorption to amorphous carbon support films. To obtain the initial model for cryo-EM refinement, 3D reconstructions were calculated by using the random conical tilt method (RCT)^48^. Tilted (−55°) and un-tilted image pairs were obtained under low-dose conditions and particles were selected using the TiltPicker program^49^ and montaged for interactive screening, yielding ~13,400 particles of the Tel1. We run iterative alternating rounds of supervised multi-reference alignment and classification as well as reference-free alignment to improve the homogeneity of the image classes. All the 3D reconstructions were calculated with SPIDER^50^ and SPARX.^51^

### Sample vitrification and cryo-EM data collection

Samples were diluted to final concentrations of 20–50 μg/mL (20 mM HEPES, pH 8.0, 40 mM KOAc, 5 mM MgCl_2_, 0.1% trehalose, 2 mM DTT, 0.01% NP-40) and 3 μL of aliquots were applied to freshly glow discharged Quantifoil R2/1 grids coated with a second layer of thin carbon film. The grids were blotted for 3–4 s at 4 °C in 100% humility, and then plunged into liquid ethane using a FEI Vitrobot (FEI Company). Frozen grids were stored in liquid nitrogen. The grids were firstly loaded into a Gatan 626 cryo-holder and transferred to an FEI Tecnai TF20 electron microscope to check the quality of the sample vitrification. Then, the grids were transferred to Titan Krios equipped with a field emission source and operated at 300 kV. Images were recorded on a K2 direct electron detector at a nominal magnification of 18,000× with a defocus range of −2 μm to −3 μm, resulting in a calibrated sampling of 1.35 Å per pixel. The total dose was set to be 50 electrons per Å^2^ on the specimen and the exposure time was 11.55 s. Each image was fractionated into 50 frames.

### Image processing

Frames were summed to a single micrograph for subsequent processing using motion-correction procedure described by Xueming Li^52^. The graphics processing unit (GPU)-accelerated program Gctf^53^ was used for determining the contrast transfer function parameters from the drift-corrected averaged images. At first, about 2,000 particles were manually picked to generate initial reference-free 2D class averages. The 2D class averages were used as a template for the GPU-accelerated program Gautomatch^53^. Finally, 221,126 particles were picked after manually cleaning and several rounds of 2D classification. 2D and 3D classifications and auto-refinement were performed using RELION2.0 (beta)^54^. The RCT reconstruction was low-pass filtered to 60 Å and used as the starting model for 3D classification. The 3D classification with six classes was performed without symmetry (C1). Two classes with symmetrical features (class 5 and class 6) were combined and subjected to further 2D classification and particle sorting. The resulting 83,185 particles were subjected to 3D refinement with C2 symmetry, which used gold-standard Fourier shell correlation (FSC) calculations to avoid overfitting. The postprocessing was carried out by “postprocess procedure” in Relion using a provided B-factor of ‒169.6. The final reconstruction was calculated at resolutions of 4.1 Å based on the gold-standard FSC of 0.143 criterion. The other 4 classes with asymmetric features were then combined for 2D classification and particle sorting, and the resulting 131,656 particles were subjected to further 3D classifications. A final set of particles (class 2, 51,010 particles) were subjected to 3D refinement without symmetry, generating a 4.3 Å resolution map after postprocessing. We further performed a focused 3D classification on the class 2 using a soft edged monomer mask. The dominate class (class 4, 25,708 particles) was subjected to ‘auto-refinement’ and ‘postprocessing’ procedures, generating a 4.3 Å resolution reconstruction with improved structural features. Local resolution map was calculated using ResMap^55^. All the 3D structures were displayed by Chimera^56^.

### Model building and simulation

The FATKIN domain (1,040 aa) and the Pincer domain (619 aa) of Tel1 were de novo model built using COOT^57^. Primary sequence assignment was guided mainly by the clearly resolved bulky residues such as Phe, Tyr, Trp, Lys and Arg. During model building, secondary structural predication was used for tracing the main chains. A model of the Spiral region of Tel1 was generated based on the model of cryo-EM structure of human ATM (PDBID: 5NP0)^17^ using SWISS MODE^58^. Rigid body refinement was then performed in COOT using Jiggle fit. The model of the Spiral domain generally matches with the electron density map and some mismatched regions were then manually adjusted in COOT according to the EM density. Real-space refinement (phenix.real_space_refine) in Phenix^59^ was used for model refinement with secondary structure and stereochemical constraints applied. The refined model was then further adjusted in COOT.

### Data availability

The 3D cryo-EM density maps reported in this paper have been deposited in the EM Databank under accession numbers: EMD-9893 (symmetric dimer), EMDB-9894 (asymmetric dimer) and EMDB-9892 (compact monomer). The corresponding models in the Protein Data Bank as PDB IDs: 6JXC (symmetric dimer) and 6JXA (compact monomer).

## Supplementary information

Supplementary information, Figure S1

Supplementary information, Figure S2

Supplementary information, Figure S3

Supplementary information, Figure S4

Supplementary information, Figure S5

Supplementary information, Figure S6

Supplementary information, Figure S7

Supplementary information, Figure S8

Supplementary information, Figure S9

Supplementary information, Figure S10

Supplementary information, Figure S11

Supplementary information, Figure S12

Supplementary information, Figure S13

Supplementary information, Figure S14

Supplementary information, Figure S15

Supplementary information, Figure S16

Supplementary information, Figure S17

Supplementary information, Figure S18

Supplementary information, Figure S19

Supplementary information, Figure S20

Supplementary information, Figure S21

Supplementary information, Table S1
