# Supplementary material for: Structural basis of allosteric regulation of Tel1/ATM kinase
Source: Cell Res. 2019 May 16;29(8):655–65. doi: 10.1038/s41422-019-0176-1 (PMC6796912; doi:10.1038/s41422-019-0176-1)
Supplement: Supplementary file 1 — Supplementary information, Figure S1 [file 41422_2019_176_MOESM1_ESM.pdf]

Supplementary information, Fig. S1

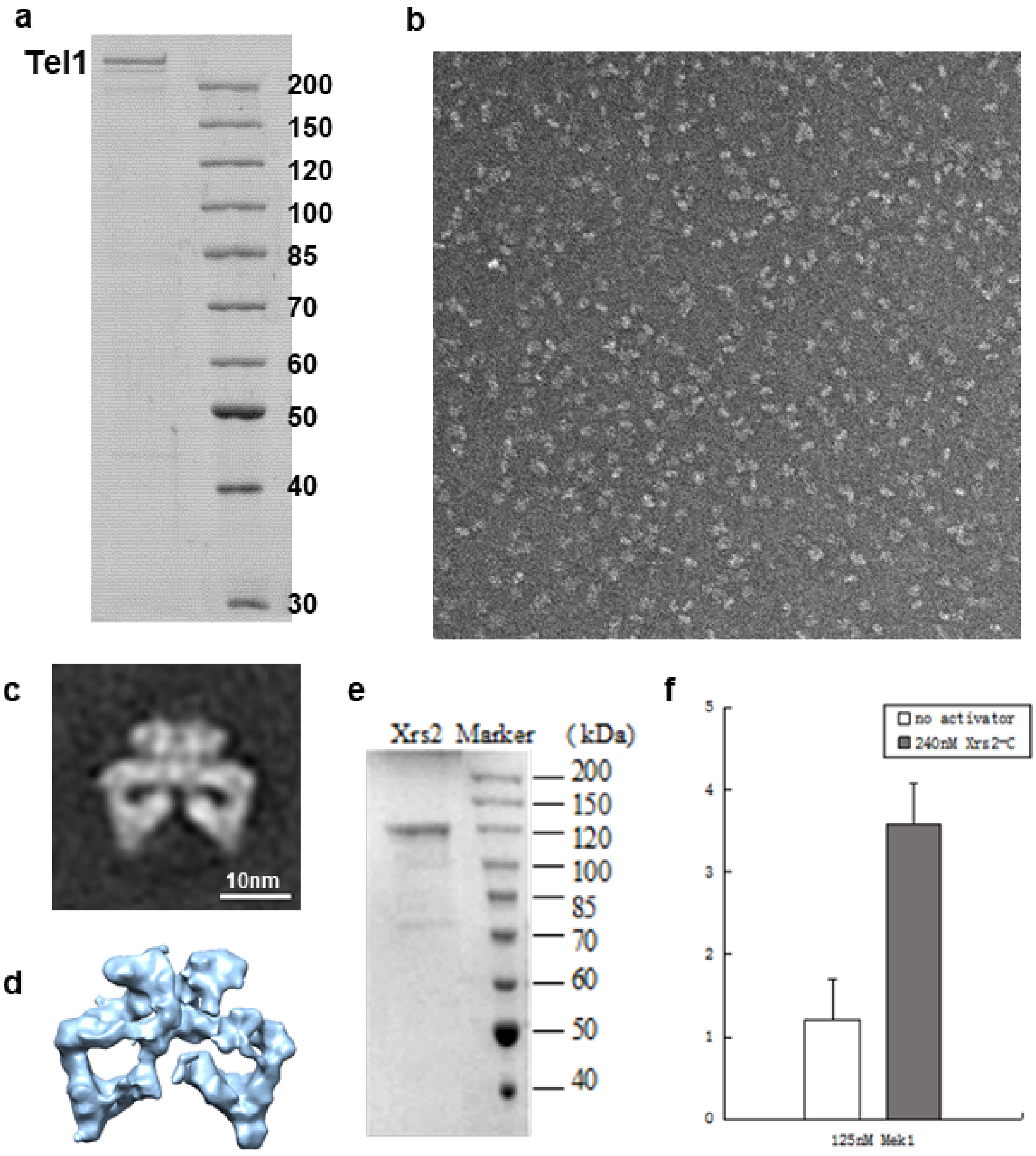

Fig. S1 Purification and preliminary EM analysis of the Tel1 kinase. **a** SDS-PAGE analysis of the Tel1 kinase endogenously purified from *S. cerevisiae*. **b** A typical electron micrograph showing the negative stained Tel1 particles. **c** A typical 2D class average of Tel1 preserved under negative stain. Scale bar, 100 Å. **d** The initial model of Tel1 calculated by the random conical tilt (RCT) method. **e** SDS-PAGE analysis of the full-length Xrs2. **f** Kinase activity assays comparing the basal and stimulated activity.
