# Supplementary material for: Structural basis of allosteric regulation of Tel1/ATM kinase
Source: Cell Res. 2019 May 16;29(8):655–65. doi: 10.1038/s41422-019-0176-1 (PMC6796912; doi:10.1038/s41422-019-0176-1)
Supplement: Supplementary file 2 — Supplementary information, Figure S2 [file 41422_2019_176_MOESM2_ESM.pdf]

Supplementary information, Fig. S2

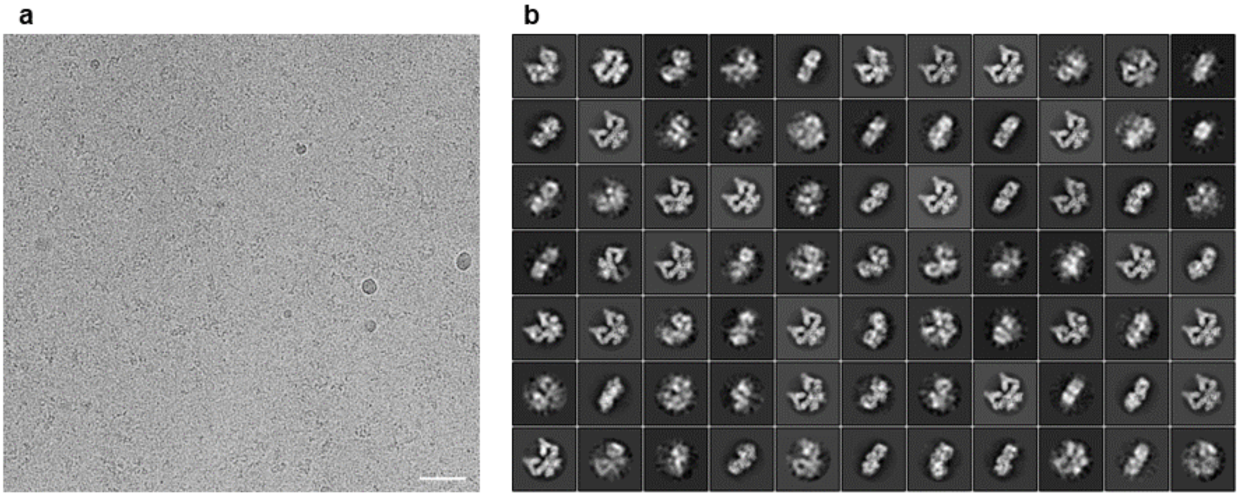

Fig. S2 Preliminary cryo-EM analysis of the Tel1 kinase. **a** A typical micrograph of Tel1 preserved in vitrified ice. Scale bar, 50 nm. **b** Typical 2D class averages obtained after reference-free alignment and classification of images of Tel1 particles preserved in vitrified ice.
