# Supplementary material for: Structural basis of allosteric regulation of Tel1/ATM kinase
Source: Cell Res. 2019 May 16;29(8):655–65. doi: 10.1038/s41422-019-0176-1 (PMC6796912; doi:10.1038/s41422-019-0176-1)
Supplement: Supplementary file 3 — Supplementary information, Figure S3 [file 41422_2019_176_MOESM3_ESM.pdf]

Supplementary information, Fig. S3

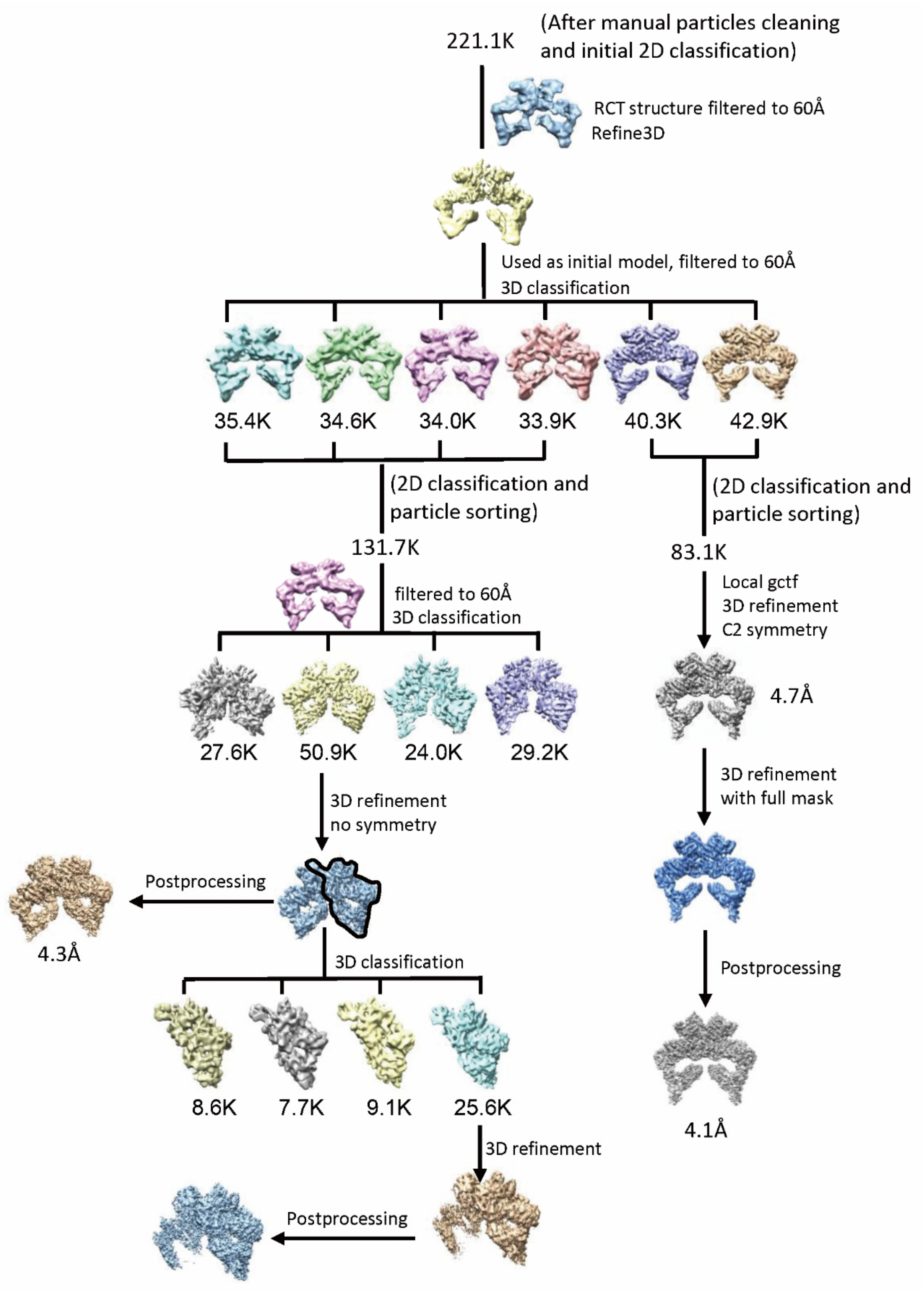

Fig. S3 Cryo-EM reconstruction of the Tel1 homodimer. The schematic diagram of the procedures is shown. Details are provided in Materials and Methods.
