# Supplementary material for: Structural basis of allosteric regulation of Tel1/ATM kinase
Source: Cell Res. 2019 May 16;29(8):655–65. doi: 10.1038/s41422-019-0176-1 (PMC6796912; doi:10.1038/s41422-019-0176-1)
Supplement: Supplementary file 4 — Supplementary information, Figure S4 [file 41422_2019_176_MOESM4_ESM.pdf]

Supplementary information, Fig. S4

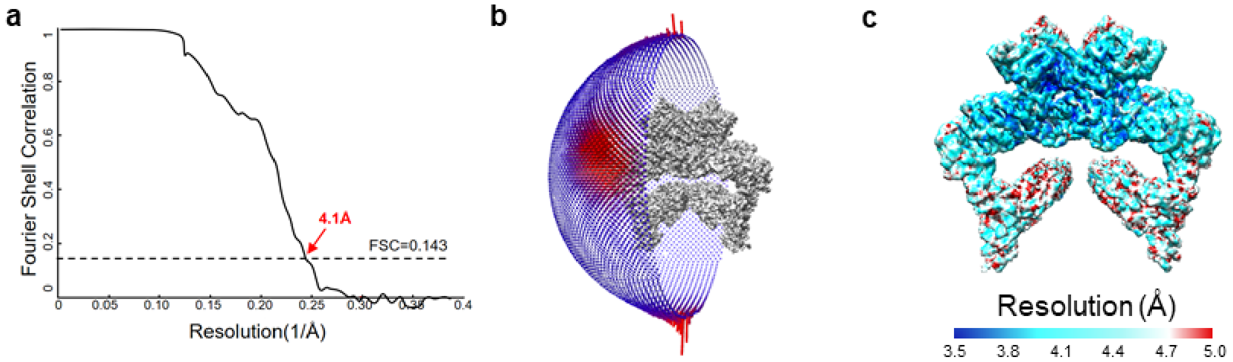

Fig. S4 Cryo-EM reconstruction of the Tel1 symmetric dimer. **a** FSC curve for the cryo-EM density map according to the gold-standard criterion. The final resolution is 4.1 Å. **b** Angular distribution for the final reconstruction of the Tel1 symmetric dimer. Each cylinder represents one view and the height of the cylinder is proportional to the number of particles for that view. **c** 3D density map colored according to local resolution estimated by ResMap.
