# Supplementary material for: Structural basis of allosteric regulation of Tel1/ATM kinase
Source: Cell Res. 2019 May 16;29(8):655–65. doi: 10.1038/s41422-019-0176-1 (PMC6796912; doi:10.1038/s41422-019-0176-1)
Supplement: Supplementary file 5 — Supplementary information, Figure S5 [file 41422_2019_176_MOESM5_ESM.pdf]

Supplementary information, Fig. S5

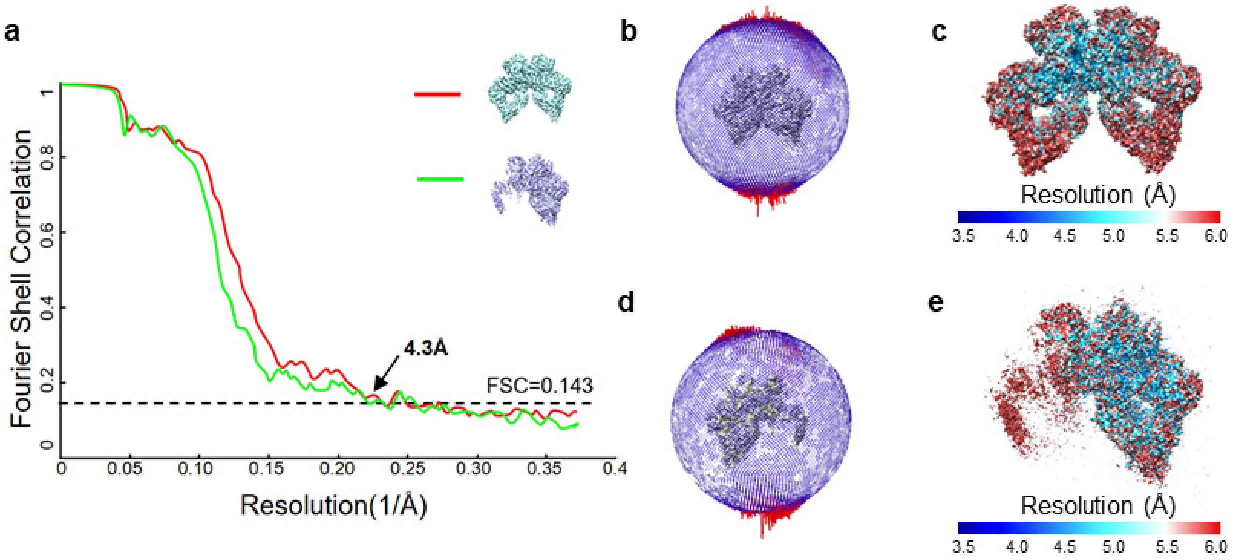

Fig. S5 Cryo-EM reconstruction of the Tel1 asymmetric dimer. **a** FSC curves for the cryo-EM density maps of asymmetric dimer and compact monomer according to the gold-standard criterion. The final resolution is 4.3 Å. **b** Angular distribution for the final reconstruction of the Tel1 asymmetric dimer. Each cylinder represents one view and the height of the cylinder is proportional to the number of particles for that view. **c** 3D density map of Tel1 asymmetric dimer colored according to local resolution estimated by ResMap. **d** Angular distribution for the final reconstruction of the Tel1 compact monomer. Each cylinder represents one view and the height of the cylinder is proportional to the number of particles for that view. **e** 3D density map of Tel1 compact monomer colored according to local resolution estimated by ResMap.
