# Supplementary material for: Structural basis of allosteric regulation of Tel1/ATM kinase
Source: Cell Res. 2019 May 16;29(8):655–65. doi: 10.1038/s41422-019-0176-1 (PMC6796912; doi:10.1038/s41422-019-0176-1)
Supplement: Supplementary file 7 — Supplementary information, Figure S7 [file 41422_2019_176_MOESM7_ESM.pdf]

Supplementary information, Fig. S7

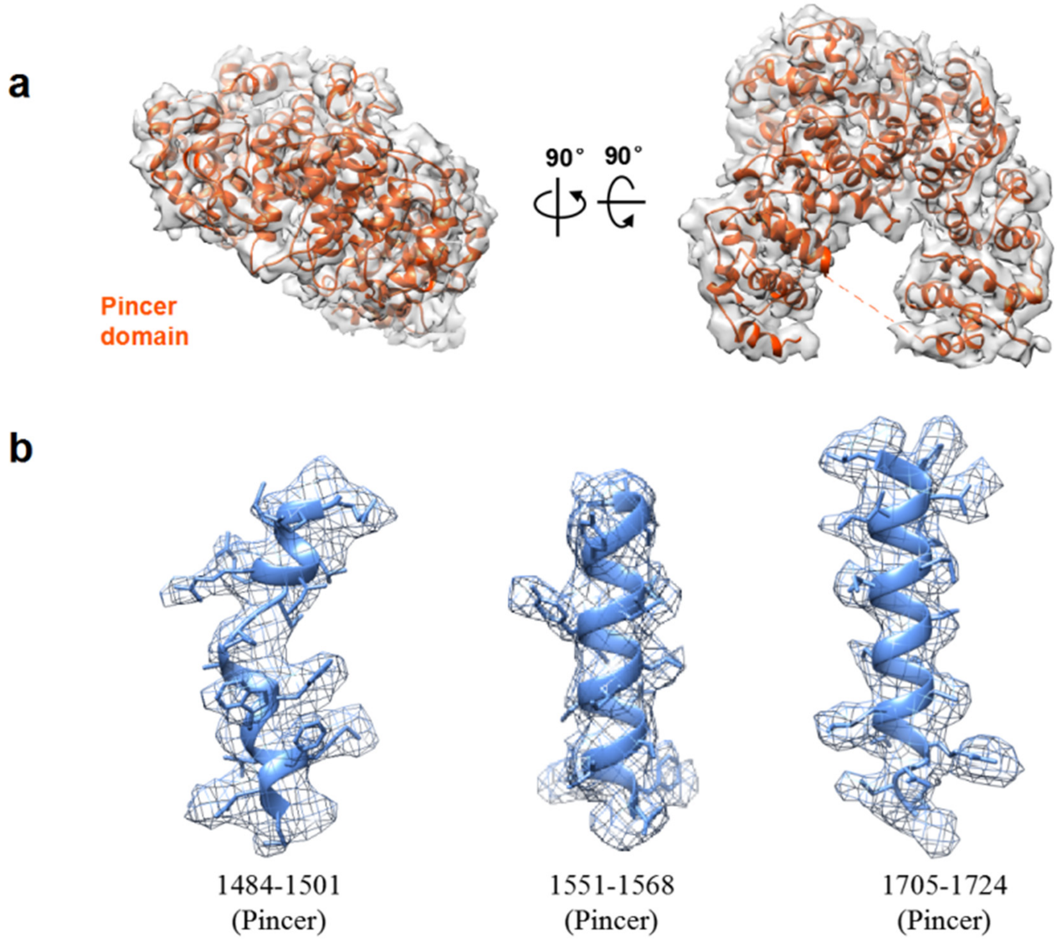

Fig. S7 The close-up views of the model-map fitting and side-chains in Pincer domain of Tel1 symmetric dimer. **a** Three close-up views of the model-map fitting in Pincer domain. **b** EM maps of representative α-helices of Tel1 Pincer domain are shown as blue meshes. The corresponding models are shown as blue sticks.
