# Supplementary material for: Structural basis of allosteric regulation of Tel1/ATM kinase
Source: Cell Res. 2019 May 16;29(8):655–65. doi: 10.1038/s41422-019-0176-1 (PMC6796912; doi:10.1038/s41422-019-0176-1)
Supplement: Supplementary file 8 — Supplementary information, Figure S8 [file 41422_2019_176_MOESM8_ESM.pdf]

Supplementary information, Fig. S8

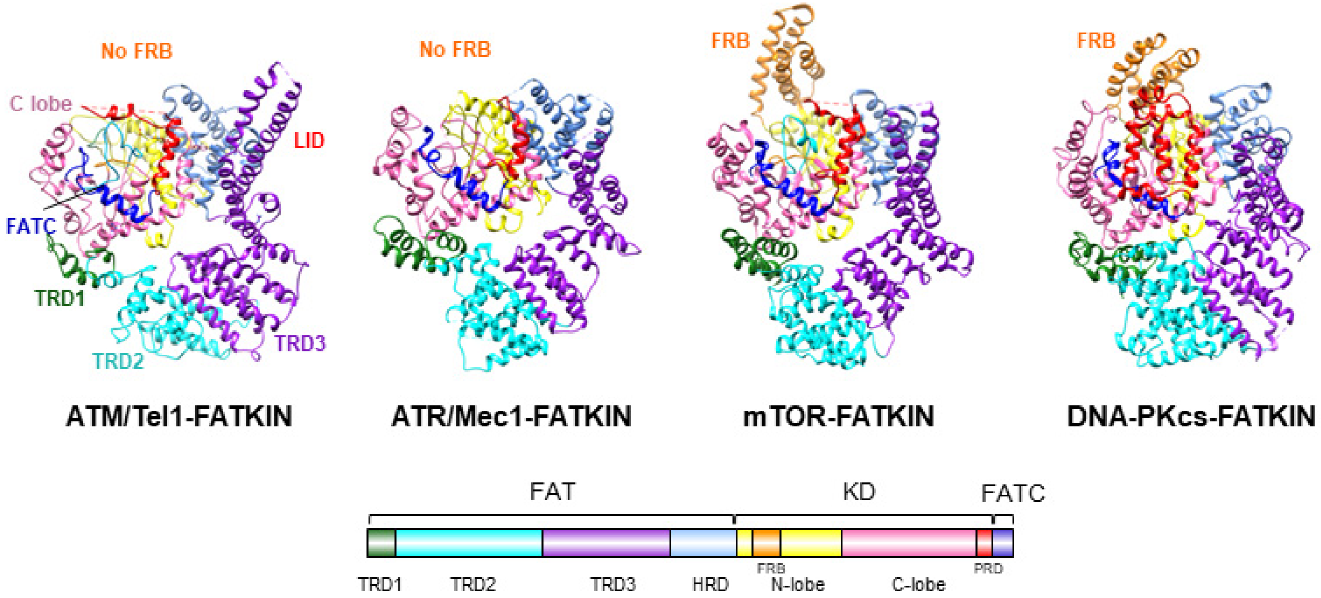

Fig. S8 Structural comparison of the FATKIN of the Tel1 with that of ATR/Mec1, mTOR and DNA-PKcs. The FATKIN of Tel1, ATR/Mec1 (PDB ID: 5X6O)[14], mTOR (PDB ID: 4JSV)[12] and DNA-PKcs (PDB ID: 5LUQ)[10] are color-coded by domain assignment. The domain organization and color scheme are shown at bottom.
