# Supplementary material for: Structural basis of allosteric regulation of Tel1/ATM kinase
Source: Cell Res. 2019 May 16;29(8):655–65. doi: 10.1038/s41422-019-0176-1 (PMC6796912; doi:10.1038/s41422-019-0176-1)
Supplement: Supplementary file 9 — Supplementary information, Figure S9 [file 41422_2019_176_MOESM9_ESM.pdf]

Supplementary information, Fig. S9

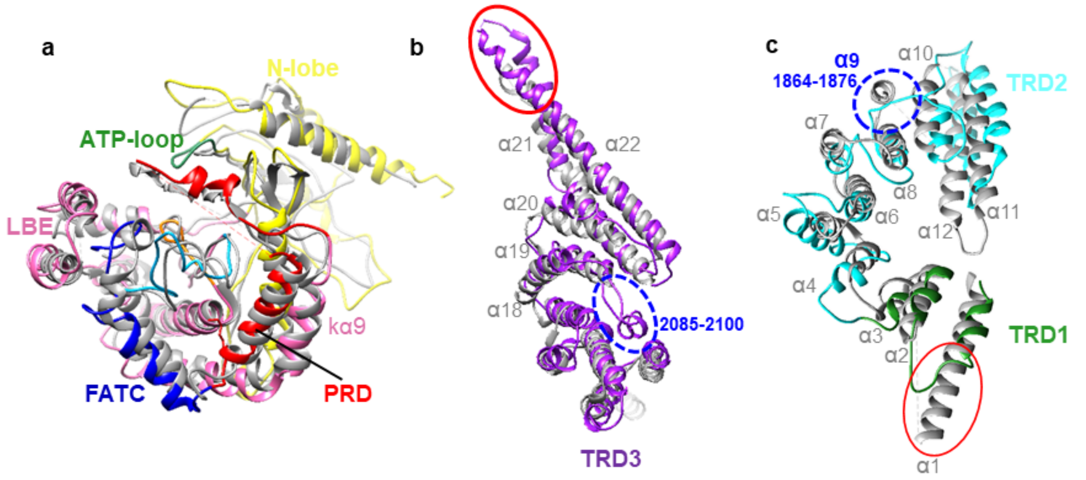

Fig. S9 Structure comparison of the catalytic core of the yeast Tel1 with that of the human ATM. **a** The structural model of yeast Tel1 kinase domain (color-coded as Supplementary information, Fig. S8) was aligned with that of human ATM model (gray, PDB ID: 5NP0)[17]. **b** The TRD3 domain of the yeast Tel1 (colored in purple) was aligned with that of human ATM (gray). The red ellipse highlights the yeast LID domain (α21 and α22) is longer than the human counterpart. The blue dashed ellipse highlights the extra helix (2,085-2,100) in yeast Tel1 compared with human ATM of TRD3. **c** The TRD1 (forest green) and TRD2 (cyan) domains of the yeast Tel1 were aligned with the human counterparts (gray). The red ellipse denotes the α1 helix in human ATM is longer than that in yeast Tel1. The blue dashed ellipse highlights the extra helix in human ATM compared with yeast Tel1 of TRD2.
