# Supplementary material for: Structural basis of allosteric regulation of Tel1/ATM kinase
Source: Cell Res. 2019 May 16;29(8):655–65. doi: 10.1038/s41422-019-0176-1 (PMC6796912; doi:10.1038/s41422-019-0176-1)
Supplement: Supplementary file 10 — Supplementary information, Figure S10 [file 41422_2019_176_MOESM10_ESM.pdf]

Supplementary information, Fig. S10

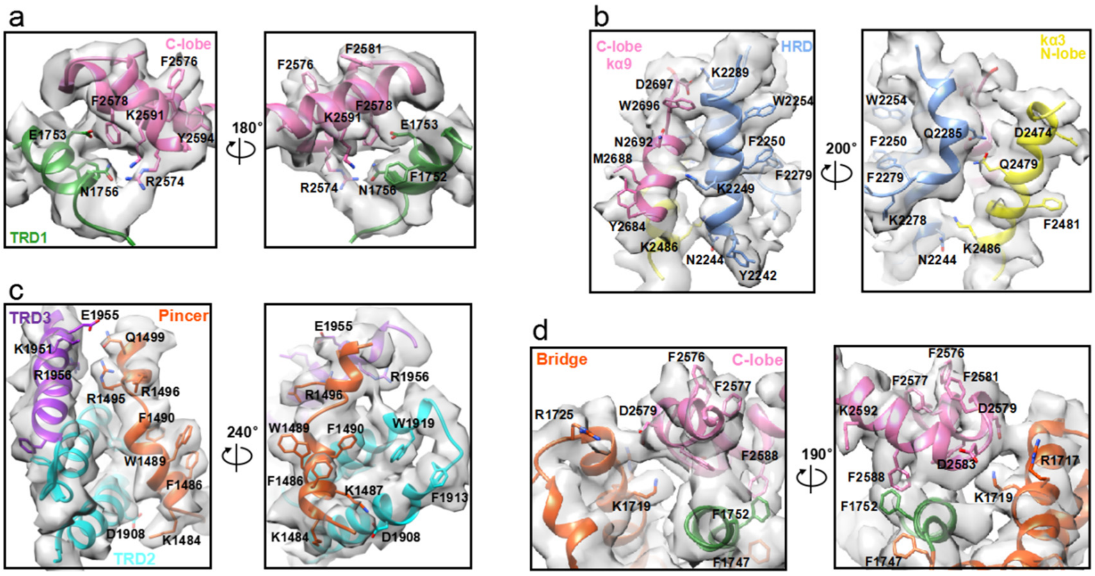

Fig. S10 The close-up views of the model-map fitting of the intramolecular interfaces. **a, b** Close-up views of the model-map fitting of the interactions between FAT and kinase domains (Related to Fig. 2a). **c, d** Close-up views of the model-map fitting of the interactions between Pincer and FATKIN domains (Related to Fig. 2b).
