# Supplementary material for: Structural basis of allosteric regulation of Tel1/ATM kinase
Source: Cell Res. 2019 May 16;29(8):655–65. doi: 10.1038/s41422-019-0176-1 (PMC6796912; doi:10.1038/s41422-019-0176-1)
Supplement: Supplementary file 11 — Supplementary information, Figure S11 [file 41422_2019_176_MOESM11_ESM.pdf]

Supplementary information, Fig. S11

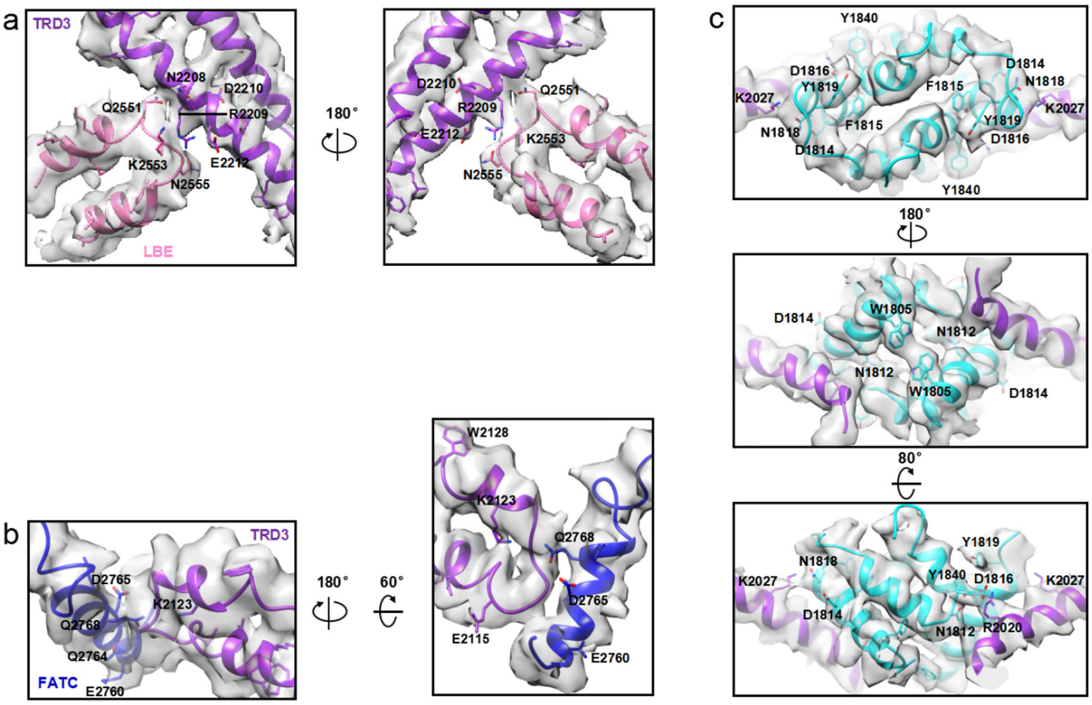

Fig. S11 The close-up views of the model-map fitting of the intermolecular interfaces (Related to Figs. 2d and 3b). **a** Close-up views of the model-map fitting of the intermolecular LID-LBE (Related to Figs. 2d, left panel and 3b). **b** Close-up views of the model-map fitting of the intermolecular TRD3-FATC (Related to Fig. 2d, middle panel). **c** Close-up views of the model-map fitting of the intermolecular TRD2-TRD2/TRD3-TRD2 interfaces (Related to Fig. 2d, right panel).
