# Supplementary material for: Structural basis of allosteric regulation of Tel1/ATM kinase
Source: Cell Res. 2019 May 16;29(8):655–65. doi: 10.1038/s41422-019-0176-1 (PMC6796912; doi:10.1038/s41422-019-0176-1)
Supplement: Supplementary file 12 — Supplementary information, Figure S12 [file 41422_2019_176_MOESM12_ESM.pdf]

Supplementary information, Fig. S12

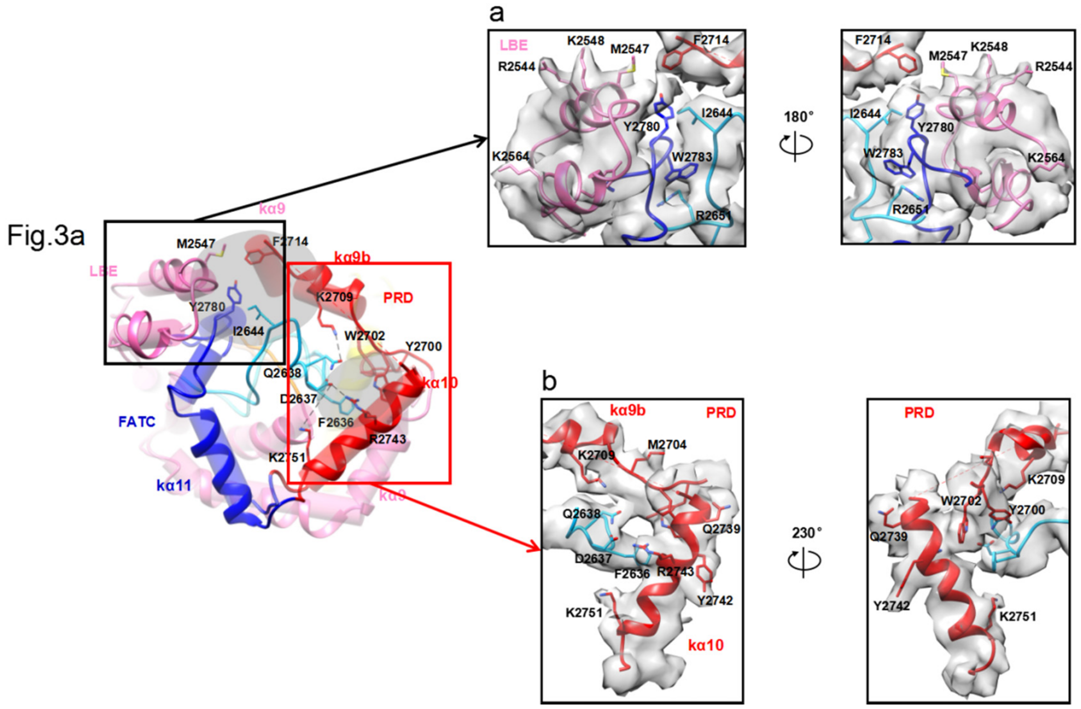

Fig. S12 The close-up views of the model-map fitting of the substrate binding groove (Related to Fig. 3a).
