# Supplementary material for: Structural basis of allosteric regulation of Tel1/ATM kinase
Source: Cell Res. 2019 May 16;29(8):655–65. doi: 10.1038/s41422-019-0176-1 (PMC6796912; doi:10.1038/s41422-019-0176-1)
Supplement: Supplementary file 13 — Supplementary information, Figure S13 [file 41422_2019_176_MOESM13_ESM.pdf]

Supplementary information, Fig. S13

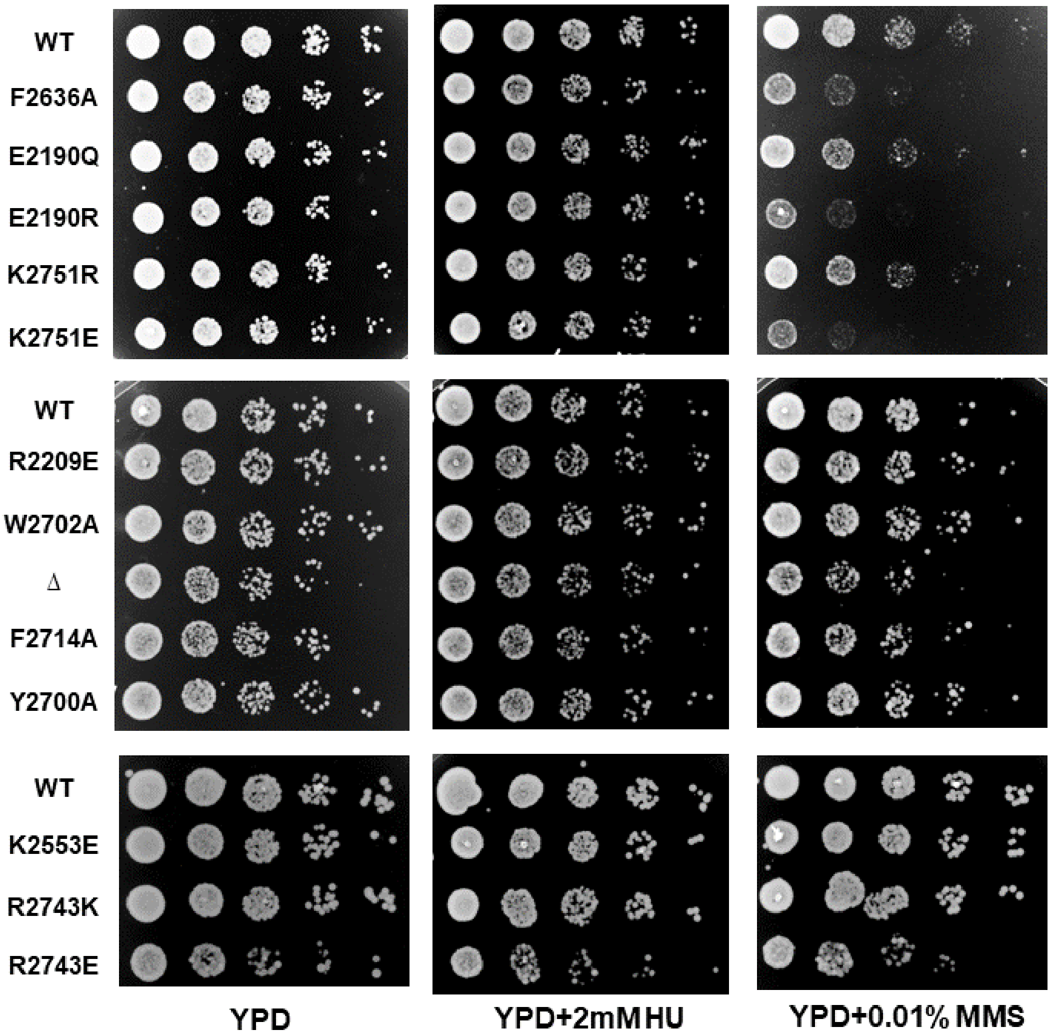

Fig. S13 DNA damage sensitivity of yeast strains containing WT and mutant Tel1. Exponential yeast cultures were serially diluted and spotted onto YPD plates supplemented with 2 mM HU or 0.01% MMS. The plates were incubated at 30 °C for 2 days.
