# Supplementary material for: Structural basis of allosteric regulation of Tel1/ATM kinase
Source: Cell Res. 2019 May 16;29(8):655–65. doi: 10.1038/s41422-019-0176-1 (PMC6796912; doi:10.1038/s41422-019-0176-1)
Supplement: Supplementary file 14 — Supplementary information, Figure S14 [file 41422_2019_176_MOESM14_ESM.pdf]

Supplementary information, Fig. S14

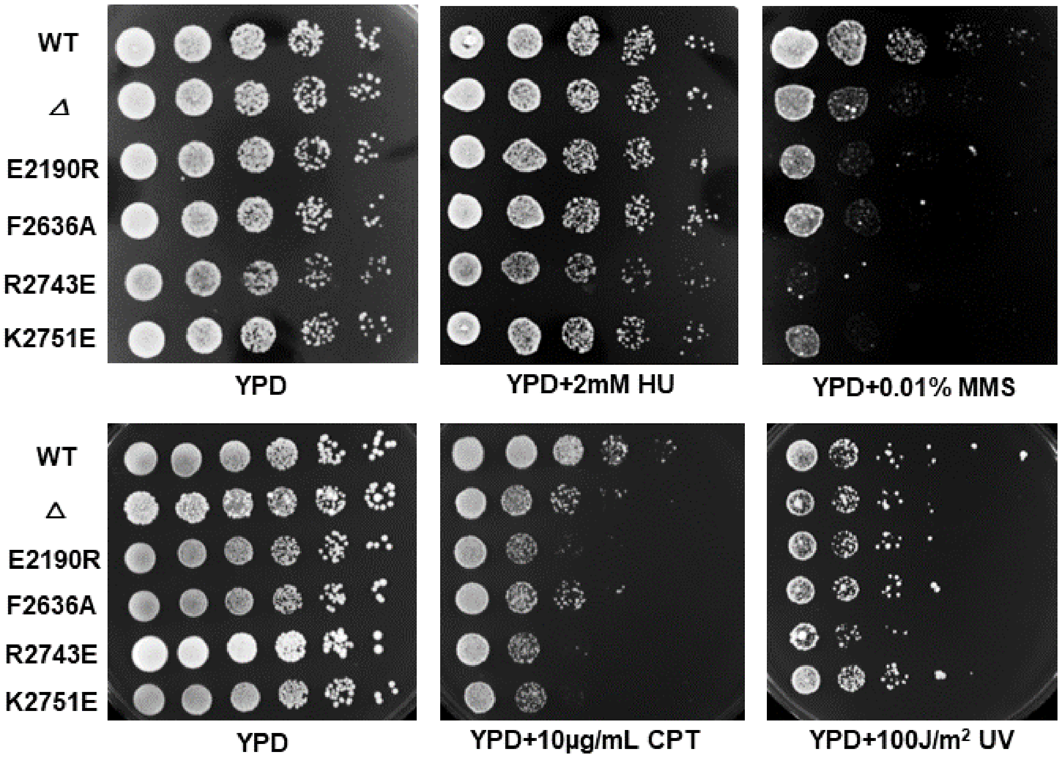

Fig. S14 DNA damage sensitivity of Tel1 mutants: Δ (truncation of LID disordered region), E2190R (within LID disordered region), F2636A (activation loop), R2743E (kα10) and K2751E (kα10). Exponential yeast cultures were serially diluted and spotted onto YPD plates supplemented with 2 mM HU, 10 μg/mL CPT or 0.01% MMS or treated with 100 J/m^2 UV after plating.
