# Supplementary material for: Structural basis of allosteric regulation of Tel1/ATM kinase
Source: Cell Res. 2019 May 16;29(8):655–65. doi: 10.1038/s41422-019-0176-1 (PMC6796912; doi:10.1038/s41422-019-0176-1)
Supplement: Supplementary file 15 — Supplementary information, Figure S15 [file 41422_2019_176_MOESM15_ESM.pdf]

# Supplementary information, Fig. S15

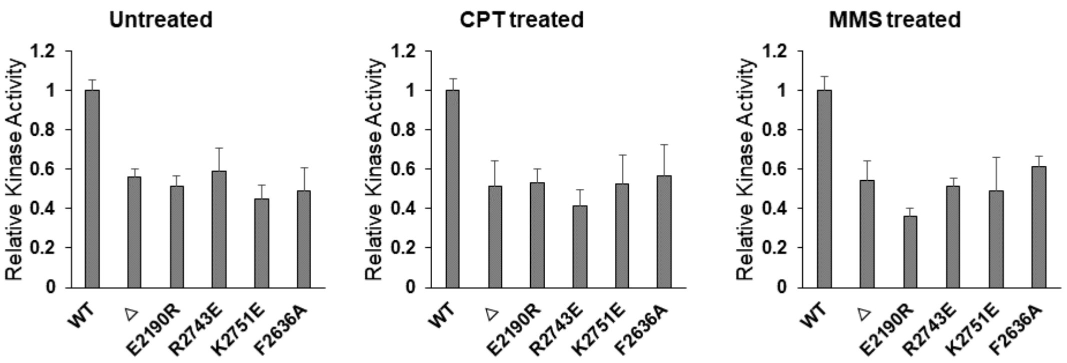


**Fig. S15** The *in vitro* kinase activity assay of WT and mutant Tel1. Results are represented as means ± SD, n = 3. The yeast cells were treated with 10 µg/mL CPT or 0.01% MMS before kinase activity measurement. The same addition amount of WT and mutant Tel1 were quantitated by western blotting.
