# Supplementary figures and images for: Structural basis of allosteric regulation of Tel1/ATM kinase
Source: Cell Res. 2019 May 16;29(8):655–65. doi: 10.1038/s41422-019-0176-1 (PMC6796912; doi:10.1038/s41422-019-0176-1)

## Supplementary information, Fig. S16

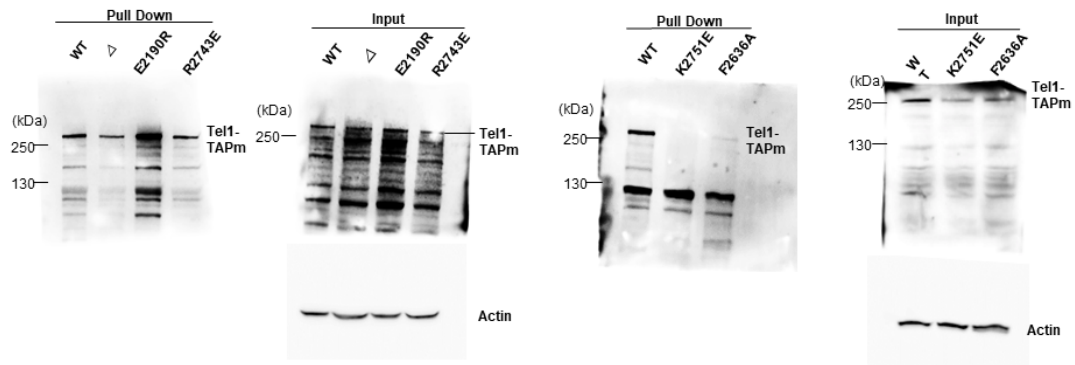

**Fig. S16** Complete western blots corresponding to Fig. 3e.

Supplement: Supplementary file 16 — Supplementary information, Figure S16 [file 41422_2019_176_MOESM16_ESM.pdf]
