# Supplementary material for: Structural basis of allosteric regulation of Tel1/ATM kinase
Source: Cell Res. 2019 May 16;29(8):655–65. doi: 10.1038/s41422-019-0176-1 (PMC6796912; doi:10.1038/s41422-019-0176-1)
Supplement: Supplementary file 17 — Supplementary information, Figure S17 [file 41422_2019_176_MOESM17_ESM.pdf]

Supplementary information, Fig. S17

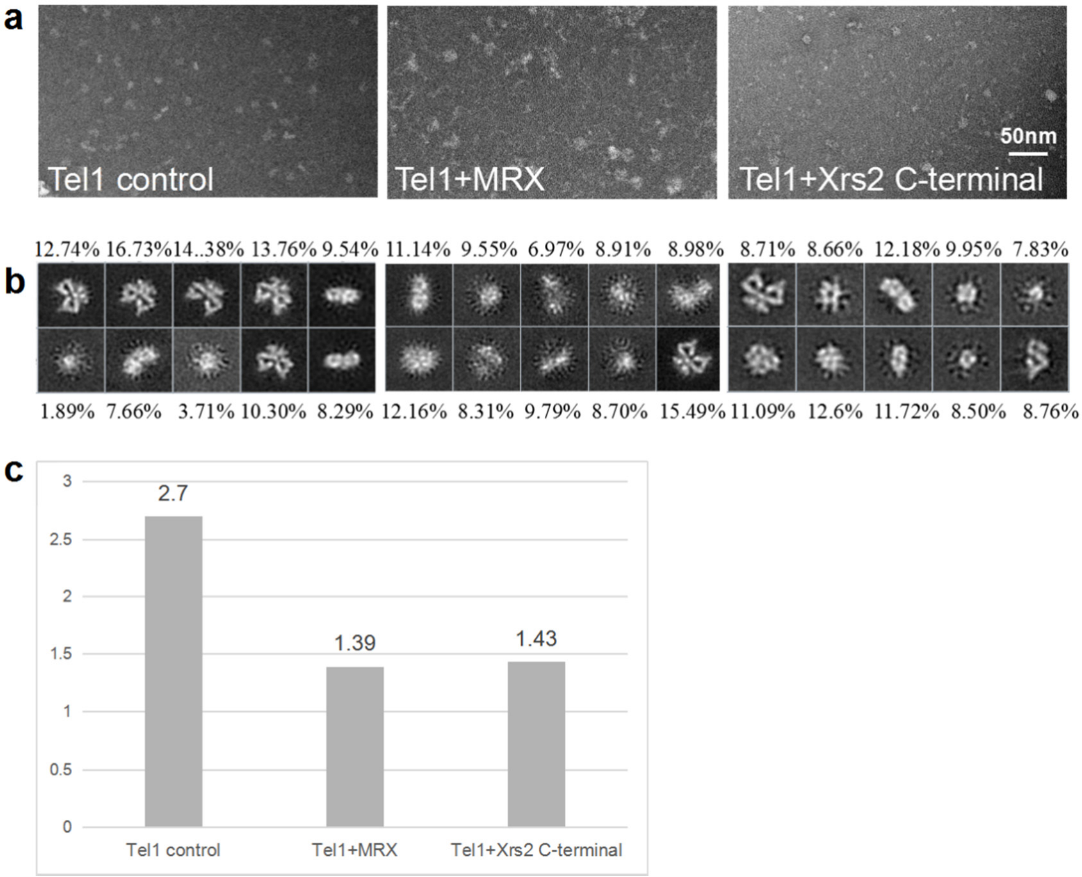

Fig. S17 Incubation with ATM/Tel1 activator prompts the distribution changes from symmetric to asymmetric conformers. **a** Negative staining EM micrographs of Tel1 and its incubation with MRX complex and Xrs2 C-terminus. Scale bar, 50 nm. **b** The corresponding 2D class averages. **c** Relative ratio of butterfly and compact forms.
