# Supplementary material for: Structural basis of allosteric regulation of Tel1/ATM kinase
Source: Cell Res. 2019 May 16;29(8):655–65. doi: 10.1038/s41422-019-0176-1 (PMC6796912; doi:10.1038/s41422-019-0176-1)
Supplement: Supplementary file 18 — Supplementary information, Figure S18 [file 41422_2019_176_MOESM18_ESM.pdf]

Supplementary information, Fig. S18

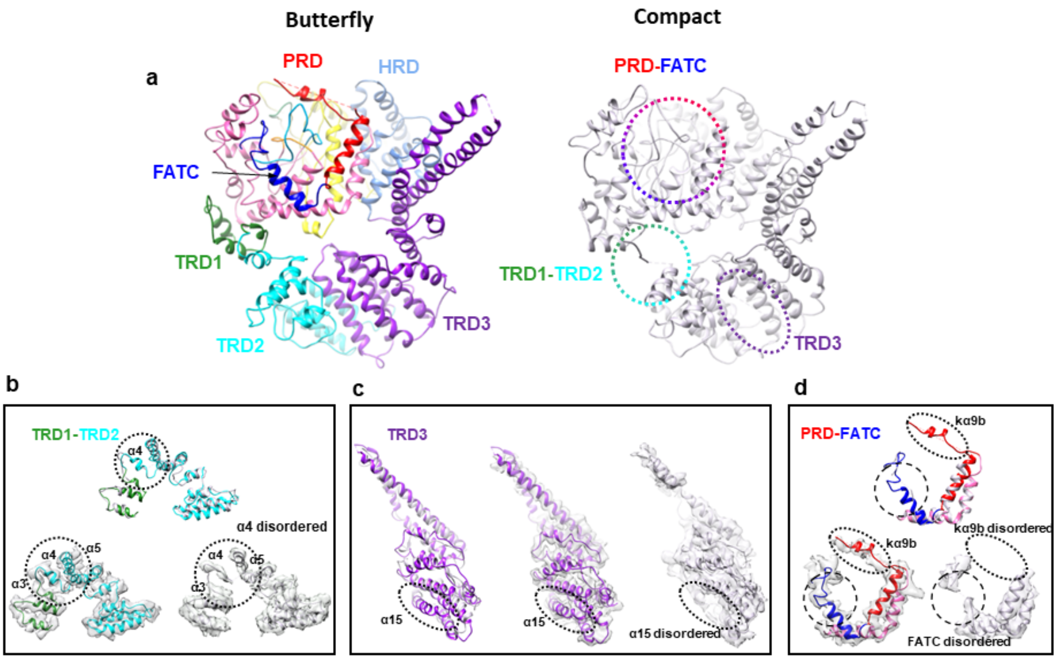

Fig. S18 Structural comparison of the disorder fragments in Tel1 Compact conformer with that of Butterfly conformer. **a** Two ribbon models of Butterfly conformer (color-coded as Supplementary information, Fig. S8) and Compact conformer (colored in gray). **a-d** The ribbon models are either directly aligned (Butterfly conformer is domain-colored and Compact conformer is in gray) or each is fitted into its own cryo-EM map. The TRD2 (α4), TRD3 (α15), PRD (kα9b) and FATC (kα11 and kα12) are largely disordered in the Compact conformer.
