# Supplementary material for: Structural basis of allosteric regulation of Tel1/ATM kinase
Source: Cell Res. 2019 May 16;29(8):655–65. doi: 10.1038/s41422-019-0176-1 (PMC6796912; doi:10.1038/s41422-019-0176-1)
Supplement: Supplementary file 19 — Supplementary information, Figure S19 [file 41422_2019_176_MOESM19_ESM.pdf]

## Supplementary information, Fig. S19

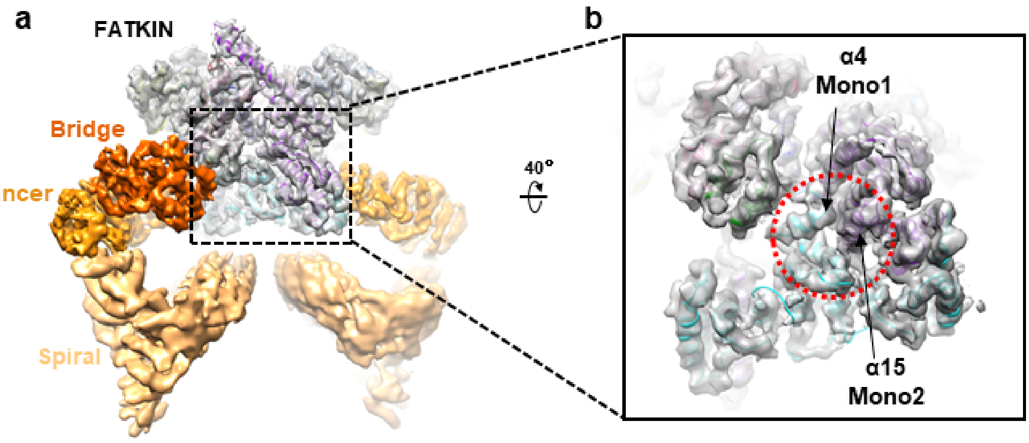


**Fig. S19** The putative substrate recruitment channel is blocked in Tel1 symmetric dimer. **a** The symmetric dimer structure denoting the location of the putative substrate recruitment channel. **b** Enlarged view of the putative substrate recruitment channel. The α4 in TRD2 of monomer 1 and α15 in TRD3 of monomer 2 are highlighted. The α15 in TRD3 directly packs against α4 in TRD2 in the symmetric dimer and stabilizes the closure of the putative substrate recruitment channel. The disordered α15 could facilitate the conformational changes in α4 in TRD2.
