# Supplementary material for: Structural basis of allosteric regulation of Tel1/ATM kinase
Source: Cell Res. 2019 May 16;29(8):655–65. doi: 10.1038/s41422-019-0176-1 (PMC6796912; doi:10.1038/s41422-019-0176-1)
Supplement: Supplementary file 20 — Supplementary information, Figure S20 [file 41422_2019_176_MOESM20_ESM.pdf]

Supplementary information, Fig. S20

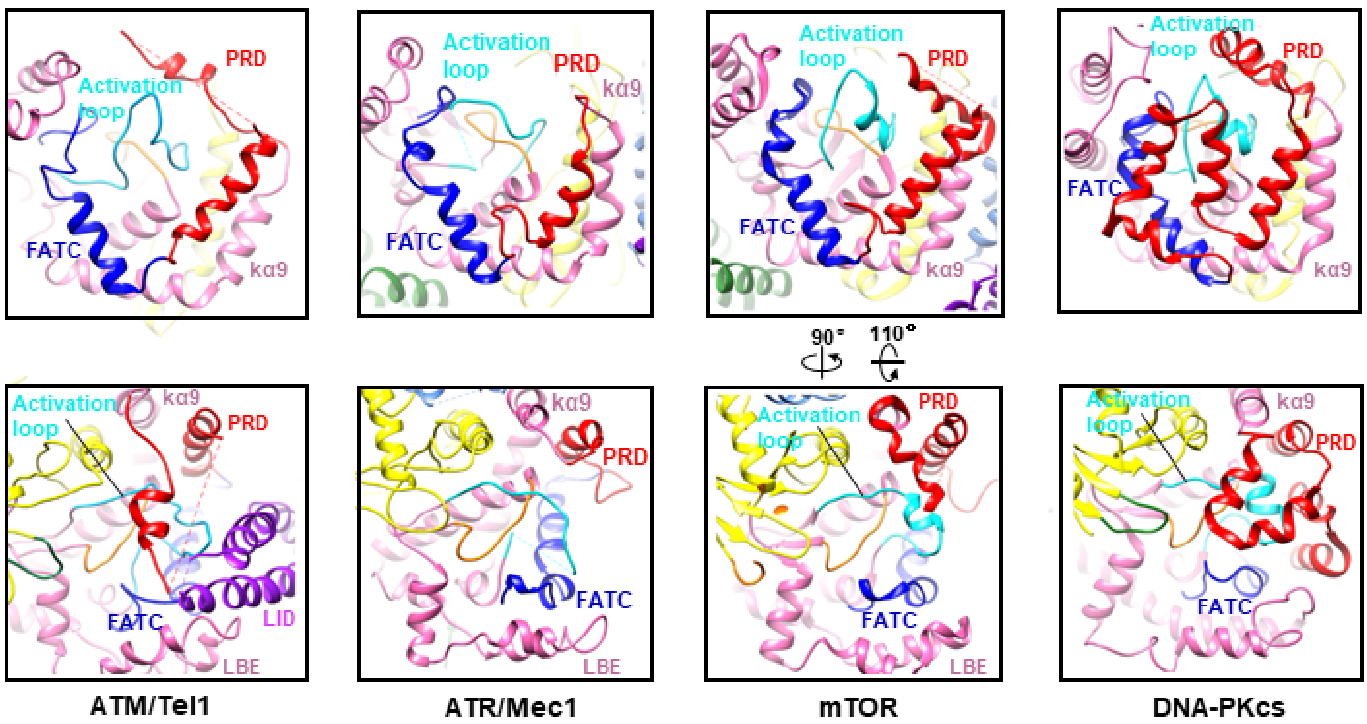

Fig. S20 Structural comparison of the substrate binding groove of the Tel1 with that of ATR/Mec1[14], mTOR[12] and DNA-PKcs[10]. Two detailed views are rotated as indicated. The kinase with the N-lobe is shown in yellow, the C-lobe is in hot pink, PRD is in red and FATC is in blue. The activation loop is colored in cyan and the catalytic loop in orange. The comparison suggests a conserved regulatory mechanism in PIKKs, which is PRD and FATC critically control substrate binding groove by immobilizing activation loop.
