# Supplementary material for: Structural basis of allosteric regulation of Tel1/ATM kinase
Source: Cell Res. 2019 May 16;29(8):655–65. doi: 10.1038/s41422-019-0176-1 (PMC6796912; doi:10.1038/s41422-019-0176-1)
Supplement: Supplementary file 21 — Supplementary information, Figure S21 [file 41422_2019_176_MOESM21_ESM.pdf]

Supplementary information, Fig. S21

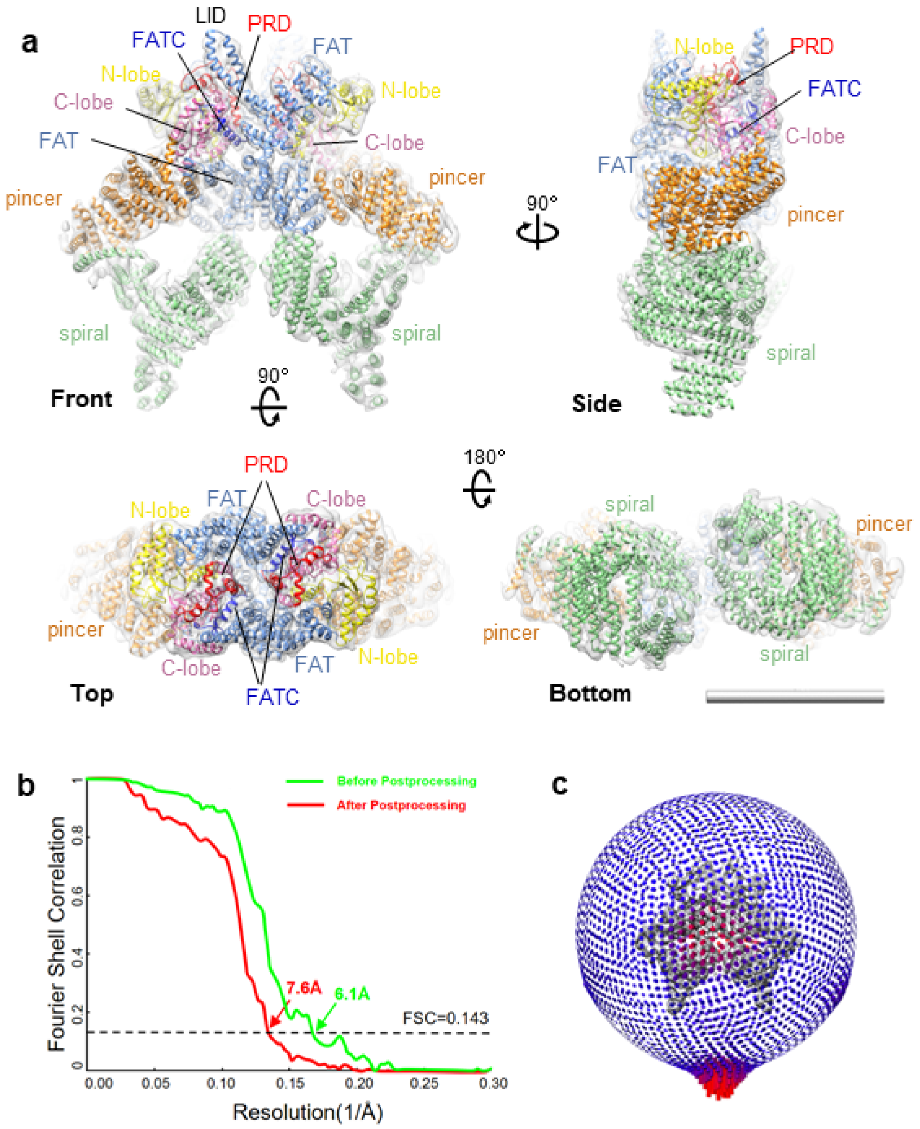

Fig. S21 Improved cryo-EM reconstruction of the *S. pombe* Tel1 kinase. **a** Different views of cryo-EM reconstruction of ATM/Tel1 endogenously purified from *S. pombe*. Scale bar, 100 Å. The reconstructed density is shown as a translucent surface, and the corresponding model is colored according to the domain architecture. ATM/Tel1 catalytic core in blue (FATC), hot pink (C-lobe), red (PRD), yellow (N-lobe) and

cornflower blue (FAT), and N-terminal α-solenoid in orange (pincer) and green (spiral). **b** FSC curve for the cryo-EM density map according to the gold-standard criterion. The final resolution is 6.1 Å. **c** Angular distribution for the final reconstruction of the *S. pombe* Tel1. Each cylinder represents one view and the height of the cylinder is proportional to the number of particles for that view.
