# Supplementary material for: Structural basis of allosteric regulation of Tel1/ATM kinase
Source: Cell Res. 2019 May 16;29(8):655–65. doi: 10.1038/s41422-019-0176-1 (PMC6796912; doi:10.1038/s41422-019-0176-1)
Supplement: Supplementary file 22 — Supplementary information, Table S1 [file 41422_2019_176_MOESM22_ESM.pdf]

Supplementary information, Table S1

Statistics of 3D reconstruction and model refinement

**Data collection**	
EM equipment	FEI Titan Krios
Voltage (kV)	300
Detector	Gatan K2
Pixel size (Å)	1.35
Electron dose ($e^-/Å^2$)	50
Defocus range (μm)	2.0~3.0
**Reconstruction**	
Software	RELION 2.0 (beta)
Number of total Particles	221.1K
Number of used Particles	83.1K
Symmetry	C2
Final Resolution (Å)	4.1
Map sharpening B-factor ($Å^2$)	-169.6
**Model building**	
Software	Coot
**Refinement**	
Software	Phenix, refmac
Average Fourier Shell correlation	0.741
R-factor	0.340
**Model composition**	
Protein residues	2787
Side chains	1157
**Validation**	
R.m.s deviations	
Bonds length (Å)	0.011
Bonds Angle (°)	1.307
Ramachandran plot statistics (%)	
Preferred	86.8
Allowed	10.9
Outlier	2.3
